# Effect of Zinc Supplementation vs Placebo on Mortality Risk and HIV Disease Progression Among HIV-Positive Adults With Heavy Alcohol Use: A Randomized Clinical Trial

**DOI:** 10.1001/jamanetworkopen.2020.4330

**Published:** 2020-05-08

**Authors:** Matthew S. Freiberg, Debbie M. Cheng, Natalia Gnatienko, Elena Blokhina, Sharon M. Coleman, Margaret F. Doyle, Tatiana Yaroslavtseva, Carly Bridden, Kaku So-Armah, Russell Tracy, Kendall Bryant, Dmitry Lioznov, Evgeny Krupitsky, Jeffrey H. Samet

**Affiliations:** 1 Vanderbilt Center for Clinical Cardiovascular Trials Evaluation (V-C3REATE), Cardiovascular Division, Vanderbilt University Medical Center, Nashville, Tennessee; 2 Geriatric Research Education and Clinical Center, Veterans Affairs Tennessee Valley Authority Health Care System, Nashville, Tennessee; 3 Department of Biostatistics, Boston University School of Public Health, Boston, Massachusetts; 4 Clinical Addiction Research and Education (CARE) Unit, Boston Medical Center, Boston, Massachusetts; 5 First Pavlov State Medical University of St Petersburg, St Petersburg, Russian Federation; 6 Biostatistics and Epidemiology Data Analytics Center (BEDAC), Boston University School of Public Health, Boston, Massachusetts; 7 Larner College of Medicine, Department of Pathology and Laboratory Medicine, The University of Vermont, Colchester; 8 Section of General Internal Medicine, Department of Medicine, Boston University School of Medicine, Boston, Massachusetts; 9 HIV/AIDS Research, National Institute on Alcohol Abuse and Alcoholism, National Institutes of Health, Bethesda, Maryland; 10 Research Institute of Influenza, St Petersburg, Russian Federation; 11 Department of Addictions, V.M. Bekhterev National Medical Research Center for Psychiatry and Neurology, St Petersburg, Russian Federation; 12 Department of Community Health Sciences, Boston University School of Public Health, Boston, Massachusetts

## Abstract

**Importance:**

Zinc supplementation can reduce alcohol-related microbial translocation and inflammation.

**Objective:**

To assess whether zinc supplementation reduces markers of mortality and risk of cardiovascular disease, reduces levels of inflammation and microbial translocation, and slows HIV disease progression in people with heavy alcohol use who are living with HIV/AIDS.

**Design, Setting, and Participants:**

This study is a double-blinded placebo-controlled randomized clinical trial of zinc supplementation among participants recruited from 2013 to 2015. Participants were recruited from HIV and addiction clinical and nonclinical care sites in St Petersburg, Russia. Participants were adults (aged 18-70 years) with documented HIV infection who were antiretroviral therapy–naive at baseline and had past 30-day heavy alcohol consumption. Data analysis was performed from February 2017 to February 2020.

**Intervention:**

Pharmacy-grade zinc gluconate supplementation (15 mg for men and 12 mg for women, taken daily by mouth for 18 months) was compared with a placebo.

**Main Outcomes and Measures:**

The primary outcome was mortality risk measured as a change in Veterans Aging Cohort Study (VACS) Index score between baseline and 18 months. The VACS Index scores range from 0 to 164, with higher scores indicating higher mortality risk. Secondary outcomes were change in CD4 cell count between baseline and 18 months, the assessment of cardiovascular disease risk (Reynolds Risk Score, which ranges from 0% to 100%, with higher scores indicating higher risk), and changes in inflammatory or microbial translocation biomarkers at 18 months. Adjusted linear regression analyses were performed.

**Results:**

A total of 254 participants (184 men [72%]; mean [SD] age, 34 [6] years) were enrolled in the trial; 126 were randomized to receive zinc, and 128 were randomized to receive placebo. Participants had high CD4 cell counts (mean [SD], 521 [292] cells/mm^3^), and 188 (74%) reported heavy drinking in the past week. In the main analyses, zinc supplementation did not affect changes in the VACS Index score at 18 months (change for zinc, mean [SD], 0.49 [14.6]; median [interquartile range], 0.0 [−7.0 to 6.0]; change for placebo, mean [SD], 5.5 [17.2]; median [interquartile range], 6.0 [−6.0 to 14.0]; adjusted mean difference [AMD], −4.68; 95% CI, −9.62 to 0.25; *P* = .06) or any secondary outcomes, including change in CD4 cell count (AMD, 41.8 cells/mm^3^; 95% CI, −20.3 to 103.8 cells/mm^3^; *P* = .19), Reynolds Risk Score (AMD, −0.014; 95% CI, −0.167 to 0.139; *P* = .85), interleukin-6 level (AMD, −0.13 pg/mL; 95% CI, −0.38 to 0.11 pg/mL; *P* = .30), dimerized plasmin fragment D level (AMD, −0.21 μg/mL fibrinogen equivalent units; 95% CI, −0.48 to 0.07 μg/mL fibrinogen equivalent units; *P* = .14), soluble CD14 level (AMD, −38.01 ng/mL; 95% CI, −166.90 to 90.88 ng/mL; *P* = .56), intestinal fatty acid binding protein level (AMD, 0.08 pg/mL; 95% CI, −0.07 to 0.22 pg/mL; *P* = .32), and lipopolysaccharide binding protein level (AMD, −0.09 ng/mL; 95% CI, −0.23 to 0.06 ng/mL; *P* = .24). In the per-protocol analyses, zinc supplementation statistically significantly affected changes in the VACS Index score at 18 months (AMD, −7.49; 95% CI, −13.74 to −1.23; *P* = .02); however, the adherence rate to zinc supplementation was 51%.

**Conclusions and Relevance:**

Zinc supplementation did not reduce mortality risk, CD4 cell counts, cardiovascular disease risk, and levels of inflammation or microbial translocation in people with heavy alcohol use who are living with HIV/AIDS. Zinc supplementation did not change the VACS Index score but may have been limited by low adherence.

**Trial Registration:**

ClinicalTrials.gov Identifier: NCT01934803

## Key Points

**Question:**

Does zinc supplementation reduce mortality and cardiovascular disease risk, reduce levels of inflammation and microbial translocation, and slow HIV disease progression among people with heavy alcohol use who are living with HIV/AIDS?

**Findings:**

In this randomized clinical trial that included 254 participants, zinc supplementation did not change the Veterans Aging Cohort Study Index score, a surrogate marker for total mortality, or other outcomes at 18 months.

**Meaning:**

Zinc supplementation did not decrease mortality risk in people with heavy alcohol use who are living with HIV/AIDS.

## Teaser

This randomized clinical trial assesses whether zinc supplementation reduces risks of mortality and cardiovascular disease, reduces levels of inflammation and microbial translocation, and slows HIV disease progression among people with heavy alcohol use who are living with HIV/AIDS.

## Introduction

Alcohol consumption and HIV infection are important causes of microbial translocation and inflammation.^1,2^ These biological processes contribute to end organ damage and mortality among people living with HIV/AIDS (PLWHA).^3^ Heavy alcohol use is common among PLWHA.^4^ Despite known associations between heavy alcohol use and microbial translocation and inflammation, researchers have primarily focused on mitigating adverse consequences of heavy alcohol use in PLWHA by promoting alcohol cessation.^5^ However, interventions to address unhealthy alcohol use are not uniformly effective.^6^ Thus, alternative treatment strategies that mitigate the negative health impacts of alcohol use without solely relying on reduction in alcohol use are needed.

Zinc deficiency is a common condition among PLWHA and those with alcohol use disorders.^7,8,9^ Any benefit from addressing this deficiency in PLWHA who are heavy users of alcohol is not clear, despite zinc supplementation being available and well tolerated.^8,10^ Infection with HIV, heavy alcohol use, and zinc deficiency are associated with negative health outcomes via mechanisms involving reductions in intestinal wall integrity and subsequent microbial translocation and inflammation.^1,2^ Zinc supplementation is associated with reduced ethanol-associated microbial translocation in animal models,^11,12^ reduced serum biomarker levels of inflammation in HIV-uninfected people,^7^ and delayed immunologic failure among PLWHA.^13^ More recently, a pilot randomized clinical trial (RCT)^14^ among PLWHA reported that zinc supplementation was associated with lower biomarker levels of inflammation and indirect measures of microbial translocation. Thus, we conducted a double-blinded, placebo-controlled RCT to assess the efficacy of zinc supplementation for reducing risk of mortality and cardiovascular disease (CVD), slowing HIV disease progression, and decreasing levels of biomarkers of inflammation and microbial translocation among PLWHA who have recent history of heavy alcohol use.

## Methods

### Objective and Study Design

The institutional review boards of Boston University Medical Campus and First St Petersburg Pavlov State Medical University approved this study. All study participants provided written informed consent. An independent data and safety monitoring board monitored both study conduct and participant safety. This study follows the Consolidated Standards of Reporting Trials (CONSORT) reporting guideline.

The protocol for the Zinc for Inflammation and Chronic disease in HIV (ZINC) trial has been previously described and is available in Supplement 1.^15^ Briefly, ZINC was a double-blinded, placebo-controlled RCT among PLWHA with heavy alcohol use in Russia that evaluated the efficacy of zinc supplementation to (1) lower mortality risk as measured by change in Veterans Aging Cohort Study (VACS) Index (primary outcome; the VACS Index scores range from 0 to 164, with higher scores indicating higher mortality risk),^16^ (2) lower CVD risk as measured by the Reynolds Risk Score (the Reynolds Risk Score ranges from 0% to 100%, with higher scores indicating higher CVD risk),^17^ (3) slow HIV disease progression as measured by change in CD4 cell count, and (4) reduce biomarker levels of inflammation as measured by interleukin-6 (IL-6), dimerized plasmin fragment D (D-dimer), soluble CD14 (sCD14), and indirect measures of microbial translocation as measured by intestinal fatty acid binding protein (IFABP) and lipopolysaccharide binding protein (LBP).^16,18^

### Participants

We recruited 254 participants between October 2013 and June 2015 from HIV and addiction clinical and nonclinical care sites and through snowball recruitment in St Petersburg, Russia. We conducted this trial at the Laboratory of Clinical Pharmacology of Addictions at the First St Petersburg Pavlov State Medical University. The ZINC trial inclusion criteria were as follows: (1) age 18 to 70 years old; (2) documented HIV infection; (3) past 30-day heavy alcohol consumption, as defined by National Institute on Alcohol Abuse and Alcoholism risky alcohol use criteria (>4 standard drinks per day [or >14 standard drinks per week] for men and >3 drinks per day [or >7 drinks per week] for women)^19^; (4) willingness to provide 2 contacts to assist with follow-up; (5) stable address within St Petersburg or districts within 100 km of St Petersburg; (6) possession of a telephone; and (7) documentation of being antiretroviral therapy (ART)–naive at the time of enrollment. Importantly, trial participation did not preclude participants from initiating ART if prescribed by their physician during the course of follow-up. Study exclusion criteria were as follows: (1) not fluent in Russian, (2) cognitive impairment precluding informed consent, and (3) breastfeeding or being pregnant. Pregnant women were excluded because of the possible negative effects of zinc supplementation during pregnancy.^20^

### Randomization

Participants were randomized in a 1:1 ratio to zinc supplementation or placebo using block randomization stratified by sex and past 7-day heavy alcohol consumption. As a double-blinded study, all participants and clinical investigators and study personnel were unaware of participant group assignment.

### Intervention

Participants were randomly assigned to zinc supplementation or placebo. For those receiving zinc supplementation, the capsules were compounded using pharmacy-grade zinc gluconate and 50 mg of riboflavin (adherence measure). On the basis of prior work,^13^ we instructed men to take 15 mg of elemental zinc gluconate and women to take 12 mg daily by mouth with a full glass of water for 18 months.

### Control Group

Control group participants followed the same study procedures and instructions as the intervention group. Participants received a sucrose placebo with riboflavin that was identical to the zinc medication in appearance and taste.

### Participant Assessment

Participants were interviewed at 4 main study visits—baseline and 6, 12, and 18 months after enrollment—and completed shorter medication visits at 6 and 12 weeks and at 9 and 15 months after enrollment. Blood was collected for testing and storage at all 4 main study visits. Participants were assessed for possible medication adverse effects at all visits. We compensated participants for their time with 1250 rubles (approximately US $22; as of April 7, 2020, US $1 = 75.5 rubles) for the main study visits and 500 rubles (approximately US $9) for the medication visits.

Data collected during the main study visits included the following: demographic characteristics and comorbidities,^21^ the Fagerström Test for Nicotine Dependence,^22^ ART use (follow-up only),^23^ and the alcohol 30-Day Timeline Follow Back.^24^ Blood specimens were tested for the following at each study visit: hemoglobin, platelets, CD4 cell count, HIV load, high sensitivity C-reactive protein, creatinine, aspartate aminotransferase, alanine aminotransferase, and 5 circulating biomarkers (sCD14, D-dimer, IL-6, IFABP, and LBP). The following laboratory testing was conducted at baseline and 18 months only: hepatitis C virus antibody and qualitative viral load, total and high-density lipoprotein cholesterol, and zinc levels. Zinc levels were tested in batches; thus, the study team was not aware of zinc level results in real time.

With regard to the 5 circulating biomarkers, all samples were measured in duplicate, and coefficients of variations (CVs) greater than 15% were not reported. Kit controls and in-house plasma controls were measured, and assay-specific CVs are described here. Soluble CD14 was measured using an enzyme-linked immunosorbent assay (ELISA) (catalog no. DC140; R&D Systems Inc) with a detectable range of 10 to 3200 ng/mL, using a standard 200-fold sample dilution. Samples with high values were diluted 400-fold and reanalyzed. Three controls were used, with interassay CVs ranging from 6.1% to 6.6%. Interleukin-6 was measured using the MSD Human IL-6 Ultra-sensitive Single-Plex kit (catalog no. K151QXG-2; MesoScale Diagnostics) which uses a sandwich ELISA technique and an electrochemiluminescent detection method, with a working range of 0.05 to 1310 pg/mL. Three controls, with interassay CVs ranging from 4.2% to 11.1%, were used. Lipopolysaccharide binding protein was measured using the MSD Human LBP Assay (catalog no. K151IYC; MesoScale Diagnostics), which also uses a sandwich ELISA technique and an electrochemiluminescent detection method, with a working range of 0.085 to 1000 ng/mL. Four controls were used for quality control, with CVs ranging from 4.4% to 4.6%. D-dimer was measured using the STAR automated coagulation analyzer (catalog no. 00515; Diagnostica Stago) using an immuno-turbidometric assay (Liatest D-DI; Diagnostica Stago). Two controls, with interassay CVs ranging from 1.6% to 23.9%, were used. Intestinal fatty acid binding protein was measured using an ELISA (catalog no. DFBP20; R&D Systems Inc), with a detectable range of 156 to 10 000 pg/mL, using a standard 5-fold dilution. Four controls were used, with interassay CVs ranging from 3.4% to 5.1%.

### Adherence

Adherence to study medication was assessed at each study visit. Our primary measure of adherence (self-report) was assessed using a visual analog scale to capture proportion of daily study medication taken in the past 6 weeks.^23^ We also assessed adherence using pill counts. Riboflavin, compounded with the zinc or placebo, was a biologic measure to capture adherence. We informed participants of the riboflavin in their pills and that its presence would be checked in their urine.^25^ Because riboflavin is not a measure of long-term adherence, it was not used as our primary adherence measure.

### Primary Outcomes

The ZINC trial’s primary outcome was a change in VACS Index score, a validated surrogate measure of mortality in PLWHA and uninfected people, between baseline and 18 months.^16,18^ A higher VACS Index score conveys a higher mortality risk. The following are the components of the VACS Index: age, CD4 cell count, HIV-1 RNA level, hemoglobin level, fibrosis-4 score, estimated glomerular filtration rate, and hepatitis C virus coinfection status. We assessed the VACS Index score at baseline and at 6, 12, and 18 months.

### Secondary Outcomes

The ZINC trial had the following secondary outcomes: (1) CVD risk as assessed by the validated Reynolds Risk Score at 18 months^17^; (2) HIV disease progression, as measured by change in CD4 cell count between baseline and 18 months; and (3) biomarker levels of inflammation, altered coagulation, monocyte activation, intestinal permeability, and microbial translocation as measured by IL-6, D-dimer, sCD14, IFABP, and LBP, respectively, at 18 months.

### Statistical Analysis

The ZINC trial was conducted and analyzed according to the intention-to-treat principle. The primary analysis used adjusted linear regression controlling for the 2 randomization stratification factors: sex and past-week heavy alcohol use. We assessed model fit using model diagnostics including residual plots. To account for skewed distributions, we used the natural log transformation for the Reynolds Risk Score, IL-6, and D-dimer. We performed multiple imputation using the iterative Markov Chain Monte Carlo technique to account for missing data in the following outcomes: VACS Index score (2 participants), CD4 cell count (1 participants), Reynolds Risk Score (4 participants), IL-6 level (3 participants), D-dimer level (3 participants), sCD14 level (3 participants), IFABP level (4 participants), and LBP level (5 participants). Variables used for imputation were sex, past-week heavy alcohol use, age, and prior data on outcome variables from baseline and 6 and 12 months.

We conducted secondary analyses to assess potential effect modification by baseline past-week heavy alcohol use and zinc deficiency (zinc level <0.75 mg/L).^13^ We also conducted secondary per-protocol analyses restricting analyses to participants who were adherent (ie, self-reporting ≥80% on the visual analog scale for ≥3 study visits). We performed post-hoc analyses to test differences in mortality between groups. Time to death was initially analyzed using the log-rank test. Cox proportional hazards models adjusted for stratification factors were used to estimate hazard ratios and 95% CIs. *P* values are 2-tailed with a significance level of less than .05 unless otherwise specified. We designed ZINC to have 80% power to detect a 10-point difference in change in VACS Index score (eg, a 5-point increase in VACS Index corresponds to a 20% relative risk increase in mortality) from baseline to 18 months, assuming an SD of 25 (based on data from the VACS study) and 20% loss to follow-up. We analyzed the data with SAS statistical software version 9.4 (SAS Institute). Data analysis was performed from February 2017 to February 2020.

## Results

In the ZINC trial, participants were young (mean [SD] age, 34 [6] years), mostly male (184 participants [72%]), regular smokers (219 participants [86%]), recent heavy alcohol users (188 participants [74%] with past 7-day heavy alcohol use), and coinfected with hepatitis C virus (224 participants [88%]), with low CVD risk (10 participants [4%] self-reported increased CVD risk) and high CD4 cell counts (mean [SD], 521 [292] cells/mm^3^) (Table 1). The mean (SD) baseline VACS Index score was 27 (16), which translates into an approximately 10% risk of mortality in 5 years.

**Table 1.** Baseline Characteristics of Zinc for Inflammation and Chronic Disease in HIV Trial Participants

Characteristic	Value, mean (SD)			*P* value
	Total (N = 254)	Zinc group (n = 126)	Placebo group (n = 128)	
Male, No. (%)	184 (72)	92 (73)	92 (72)	.84
Age, y	34 (6)	34 (5)	34 (6)	.83
Education (≥9 grades), No. (%)	208 (82)	101 (80)	107 (84)	.48
Family history of heart disease, No. (%)	56 (25)	24 (21)	32 (28)	.25
Self-reported cardiovascular disease, No. (%)	10 (4)	5 (4)	5 (4)	.98
Regular smoker, No. (%)	219 (86)	106 (84)	113 (88)	.34
Blood pressure, mm Hg				
Systolic	125 (13)	125 (13)	126 (12)	.64
Diastolic	80 (9)	80 (9)	80 (8)	.59
Cholesterol, mg/dL				
Total	138 (36)	137 (36)	138 (35)	.84
High-density lipoprotein	45 (17)	45 (18)	45 (16)	.99
Creatinine, mg/dL	0.8 (0.2)	0.8 (0.2)	0.8 (0.2)	.61
Fibrosis-4 score	2.4 (6)	2.0 (2)	2.8 (7)	.25
Hepatitis C virus antibody positive, No. (%)	224 (88)	109 (87)	115 (90)	.41
Illicit substance use, No. (%)	105 (41)	52 (41)	53 (41)	.98
Hepatitis B (self-reported), No. (%)	80 (31.5)	44 (34.9)	36 (28.1)	.24
HIV viral load, median, copies/mL	25 499	19 201	33 291	.37
CD4 cell count, cells/mm^3^	521 (292)	511 (296)	530 (288)	.62
Body mass index^a^	23 (3)	23 (3)	23 (3)	.24
Heavy alcohol use (past 7 d), No. (%)^b^	188 (74)	94 (75)	94 (73)	.83
Veterans Aging Cohort Study Index score	27 (16)	27 (17)	26 (14)	.52
Reynolds Risk Score	1 (1)	1 (1)	1 (1)	.49
Interleukin-6, pg/mL	2.3 (9)	2.8 (12)	1.9 (4)	.45
Dimerized plasmin fragment D, μg/mL fibrinogen equivalent units	0.7 (1)	0.7 (1)	0.6 (1)	.27
Soluble CD14, ng/mL	2099 (592)	2092 (601)	2106 (585)	.85
Intestinal fatty acid binding protein, pg/mL	1494 (1126)	1473 (889)	1515 (1320)	.77
Lipopolysaccharide binding protein, ng/mL	4161 (2037)	4080 (1858)	4240 (2200)	.54

SI conversion factors: To convert cholesterol to mmol/L, multiply by 0.0259; creatinine to μmol/L, multiply by 88.4; dimerized plasmin fragment D to nmol/L, multiply by 5.476.

^a^ Body mass index is calculated as weight in kilograms divided by height in meters squared.

^b^ National Institute on Alcohol Abuse and Alcoholism risky alcohol use criteria: more than 4 standard drinks per day (or >14 standard drinks per week) for men and more than 3 standard drinks per day (or >7 standard drinks per week) for women.

After enrollment, 254 participants were randomized to receive either zinc supplementation (126 participants) or placebo (128 participants) for 18 months (Figure). At the 18-month follow-up visit, 69% of participants were assessed. Compared with the placebo group, those randomized to the zinc group had a smaller, but not statistically significant, increase in VACS Index score at 18 months (mean [SD] change for zinc, 0.49 [14.6]; median [interquartile range], 0 [−7.0 to 6.0]; mean [SD] change for placebo, 5.5 [17.2]; median [interquartile range], 6.0 [−6.0 to 14.0]; adjusted mean difference [AMD], −4.68; 95% CI, −9.62 to 0.25; *P* = .06) (Table 2). There was no statistically significant difference in CD4 cell counts (median [interquartile range], −116.9 [−242.3 to 39.9cells/mm^3^ for zinc vs −135.6 [−283.9 to −48.6] cells/mm^3^ for placebo; AMD, 41.8 cells/mm^3^; 95% CI, −20.3 to 103.8 cells/mm^3^; *P* = .19) or Reynolds Risk Score (median [interquartile range], −0.17 [−0.75 to 0.38] for zinc vs −0.14 [−0.95 to 0.55] for placebo; AMD, −0.014; 95% CI, −0.167 to 0.139; *P* = .85) between participants in the zinc and placebo groups (Table 2). The participants in the zinc group had reductions in all biomarker levels except IFABP compared with participants in the placebo group (Table 2).

**Figure.**
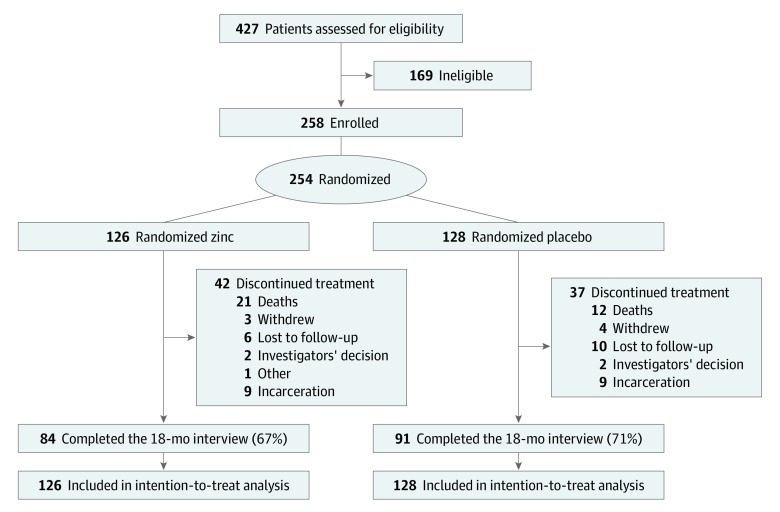
Flow of Participants Through Trial

**Table 2.** Primary and Key Secondary Outcomes: Effect of Zinc vs Placebo on Markers of Mortality, HIV Progression, Cardiovascular Disease Risk and Inflammation

Marker	Zinc group (n = 126)	Placebo group (n = 128)	Adjusted mean difference (95% CI)	*P* value
Change from baseline to 18 mo				
Veterans Aging Cohort Study Index score				
Mean (SD)	0.49 (14.6)	5.5 (17.2)	−4.68 (−9.62 to 0.25)	.06
Median (IQR)	0 (−7.0 to 6.0)	6.0 (−6.0 to 14.0)		
CD4 cell count, cells/mm^3^				
Mean (SD)	−128.8 (232.9)	−176.2 (220.6)	41.8 (−20.3 to 103.8)	.19
Median (IQR)	−116.9 (−242.3 to 39.9)	−135.6 (−283.9 to −48.6)		
At 18 mo				
Reynolds Risk Score^a^				
Mean (SD)	−0.17 (0.8)	−0.19 (0.9)	−0.014 (−0.167 to 0.139)	.85
Median (IQR)	0.17 (−0.75 to 0.38)	−0.14 (−0.95 to 0.55)		
Interleukin-6, pg/mL^a^				
Mean (SD)	−0.03 (0.9)	0.17 (0.9)	−0.13 (−0.38 to 0.11)	.30
Median (IQR)	−0.02 (−0.75 to 0.41)	0.13 (−0.43 to 0.67)		
Dimerized plasmin fragment D, μg/mL fibrinogen equivalent units^a^				
Mean (SD)	−0.94 (1.0)	−0.80 (1.0)	−0.21 (−0.48 to 0.07)	.14
Median (IQR)	−0.97 (−1.56 to −0.27)	−0.84 (−1.43 to −0.14)		
Soluble CD14, mean (SD), ng/mL	2086.26 (445.7)	2099.61 (491.8)	−38.01 (−166.90 to 90.88)	.56
Intestinal fatty acid binding protein, mean (SD), pg/mL^a^	7.19 (0.4)	7.09 (0.5)	0.08 (−0.07 to 0.22)	.32
Lipopolysaccharide binding protein, mean (SD), ng/mL^a^	8.07 (0.5)	8.20 (0.5)	−0.09 (−0.23 to 0.06)	.24

Abbreviation: IQR, interquartile range.

SI conversion factor: To convert dimerized plasmin fragment D to nmol/L, multiply by 5.476.

^a^ Natural log-transformation.

In the ZINC trial, adherence did not vary by intervention group. In prespecified secondary per-protocol analyses, including only participants who were adherent to the study medication, participants in the zinc group (64 of 126 [51%]) compared with placebo group (63 of 128 [49%]) had a statistically significant lower AMD in the VACS Index score at 18 months (−7.49; 95% CI, −13.74 to −1.23; *P* = .02) (Table 3). Of note, there was no difference in adherence by intervention group when we substituted our primary adherence measure (visual analog scale) for riboflavin (70% adherent in the zinc vs 68% in the placebo groups; difference, 2.1%; 95% CI, −11.6% to 15.8%; *P* = .76). In contrast, there were no statistically significant differences in CD4 cell count (AMD, 47.4 cells/mm^3^; 95% CI, −32.9 to 127.8 cells/mm^3^; *P* = .25), Reynolds Risk Score (AMD, −0.083; 95% CI, −0.282 to 0.116; *P* = .41), or other biomarkers, including IL-6 level (AMD, −0.13 pg/mL; 95% CI, −0.38 to 0.11 pg/mL; *P* = .30), D-dimer level (AMD, −0.21 μg/mL fibrinogen equivalent units; 95% CI, −0.48 to 0.07 μg/mL fibrinogen equivalent units; *P* = .14), soluble CD14 level (AMD, −38.01 ng/mL; 95% CI, −166.90 to 90.88 ng/mL; *P* = .56), intestinal fatty acid binding protein (AMD, 0.08 pg/mL; 95% CI, −0.07 to 0.22 pg/mL; *P* = .32), and lipopolysaccharide binding protein (AMD, −0.09 ng/mL; 95% CI, −0.23 to 0.06 ng/mL; *P* = .24) (Table 3).

**Table 3.** Secondary Per-Protocol Results: Effect of Zinc vs Placebo on Markers of Mortality, HIV Progression, Cardiovascular Disease Risk and Inflammation

Marker	Zinc group (n = 64)	Placebo group (n = 63)	Adjusted mean difference (95% CI)	*P* value
Change from baseline to 18 mo				
Veterans Aging Cohort Study Index score				
Mean (SD)	0.22 (15.2)	7.72 (16.9)	−7.49 (−13.74 to −1.23)	.02
Median (IQR)	0 (−7.0 to 6.0)	7.0 (−6.0 to 17.0)		
CD4 cell count, cells/mm^3^				
Mean (SD)	−129.5 (222.0)	−188.9 (225.0)	47.4 (−32.9 to 127.8)	.25
Median (IQR)	−90.4 (−242.3 to 12.3)	−146.2 (−295.4 to −51.0)		
At 18 mo				
Reynolds Risk Score^a^				
Mean (SD)	−0.21 (0.8)	−0.12 (0.8)	−0.083 (−0.282 to 0.116)	.41
Median (IQR)	−0.25 (−0.70 to 0.30)	−0.11 (−0.62 to 0.54)		
Interleukin-6, pg/mL^a^				
Mean (SD)	−0.02 (0.9)	0.20 (0.8)	0.012 (−0.25 to 0.28)	.93
Median (IQR)	0.06 (−0.83 to 0.57)	0.20 (−0.43 to 0.72)		
Dimerized plasmin fragment D, μg/mL fibrinogen equivalent units^a^				
Mean (SD)	−0.97 (1.0)	−0.71 (1.1)	−0.31 (−0.65 to 0.04)	.08
Median (IQR)	−0.93 (−1.56 to −0.27)	−0.71 (−1.47 to 0.06)		
Soluble CD14, mean (SD), ng/mL	2092.08 (464.2)	2149.46 (507.2)	−6.03 (−162.31 to 150.25)	.95
Intestinal fatty acid binding protein, mean (SD), pg/mL^a^	7.17 (0.4)	7.03 (0.5)	0.11 (−0.06 to 0.28)	.21
Lipopolysaccharide binding protein, mean (SD), ng/mL^a^	8.12 (0.5)	8.21 (0.5)	−0.10 (−0.27 to 0.07)	.24

Abbreviation: IQR, interquartile range.

SI conversion factor: To convert dimerized plasmin fragment D to nmol/L, multiply by 5.476.

^a^ Natural log-transformation.

In ZINC, 33 participants (13%) died before the end of the study. In post-hoc analyses, we found no statistically significant difference in mortality by zinc vs placebo study group (adjusted hazard ratio, 1.80; 95% CI, 0.88-3.65; *P* = .10). Please see the eTable in Supplement 2 for cause of death information for each study group.

Notably, no important differences in ART initiation occurred in the zinc (25.4%) vs placebo (29.7%) group (difference, 4.3%; 95% CI, −6.7% to 15.3%; *P* = .44); therefore, we do not think that ART affected the results of the trial. At the conclusion of the study, we measured zinc levels to assess zinc deficiency (ie, zinc level <0.75 mg/L) from the baseline examination, which did not vary by study group (zinc vs placebo group, 31% vs 29%; difference, 1.4%; 95% CI, −10.2% to 13.1%; *P* = .81), nor did it significantly modify the association between zinc supplementation and any of our study outcomes. Similarly, heavy alcohol use in the prior 7 days did not significantly modify the association between zinc supplementation and any of our outcomes. There were no serious adverse events that were related to study medication. Study participants reported minimal adverse effects with gastrointestinal events being most common (27 participants in total; 11 in the zinc group and 16 in the placebo group).

## Discussion

Among PLWHA with recent heavy alcohol use in the ZINC trial, participants randomized to the zinc supplementation group had a smaller mean increases in VACS Index scores between baseline and 18 months. Although this difference in change in VACS Index scores between the 2 groups was not statistically significant, it was clinically meaningful, because participants in the zinc group had a mean change of just 0.49 (corresponding to a 2% increase in mortality risk over 18 months) compared with 5.5 (corresponding to a 20% increase in mortality risk over 18 months) in the placebo group. When restricting analyses to those who were adherent to the protocol, participants in the zinc group had statistically significantly smaller increases in VACS Index scores. Although more deaths occurred in the zinc group, the difference in mortality by study group was not statistically significant. We did not detect statistically significant differences between zinc and placebo groups for CD4 cell count, Reynolds Risk Score, and IL-6, D-dimer, sCD14, IFABP, or LBP levels at 18 months.

Although prior trials have examined the potential health benefits of zinc supplementation in PLWHA, this study has multiple innovations. It is relatively large compared with previous trials of zinc^9^ and simultaneously evaluates the effects of zinc supplementation on mortality and CVD risk, as well as biomarkers reflecting disease activity. It also targets those with heavy alcohol use who may be particularly amenable to zinc’s possible beneficial effects. A recent Cochrane Review article^9^ noted 3 prior trials that included PLWHA who received zinc supplements weekly or daily and captured mortality outcomes. The authors’ conclusion was that it remains unknown “if zinc supplements have any effect on mortality” in PLWHA.^9^ Our findings in secondary analyses involving participants who were adherent, although not definitive, suggest a benefit to the VACS Index score, a surrogate measure of mortality.

Although the mechanisms for health benefits associated with zinc supplementation remain unclear, prior work suggests that zinc acts on signaling pathways involving oxidative stress and cytokine production.^26^ Among alcohol users, one hypothesis involves the oxidative stress associated with alcohol consumption and metabolism that leads to zinc deficiency by mobilizing intracellular zinc with subsequent dysfunction of tight junction proteins (eg, Z0-1 proteins) and gastrointestinal epithelial barrier dysfunction.^2^ This dysfunction, in turn, leads to microbial translocation of endotoxin, which then results in liver injury, inflammation, end organ damage, and potentially death.^2^ Our analyses involving biomarkers of inflammation and microbial translocation do not support this hypothesis. However, a recent pilot RCT of PLWHA,^14^ which did not focus on heavy alcohol users, did report that zinc supplementation was associated with lower biomarker levels of inflammation and indirect measures of microbial translocation.

### Limitations

This study has limitations that warrant discussion. First, we did not assess zinc levels before enrollment into the study because such a protocol could not be implemented practically in a real-world, resource-constrained clinical setting, and prior studies have already established that zinc deficiency is common among PLWHA and those with alcohol use disorder. Second, we could not power our trial on mortality and CVD events given our planned sample size; however, we did use validated surrogate measures of risk for mortality (VACS Index) and CVD (Reynolds Risk Score). Third, although initiation of ART could have a profound impact on all of our trial outcomes, few participants initiated ART and there was no difference in ART initiation by study group.

## Conclusions

In the ZINC trial, participants randomized to zinc supplementation had clinically meaningful, but not statistically significant, improvements in VACS Index score, a marker of mortality risk, at 18 months compared with the placebo group. Zinc supplementation did not slow HIV disease progression or reduce the risk of CVD or levels of markers of inflammation and microbial translocation. Among participants who were adherent to the study protocol, zinc supplementation statistically significantly reduced the VACS Index score, suggesting that zinc, if taken as prescribed, may reduce mortality risk in this population. On the basis of our findings as well as a recent pilot RCT that reported improvements in biomarker levels of inflammation and microbial translocation among PLWHA taking zinc supplementation, we believe that a larger trial, with greater statistical power, is warranted to determine whether zinc supplementation, a readily available, inexpensive therapy, improves health outcomes among PLWHA who are heavy alcohol users.

## Notes

Supplement 1. **Trial Protocol**

Supplement 2. **eTable.** Causes of Death in the ZINC Trial by Randomized Group (N=33)

Supplement 3. **Data Sharing Statement**

## References

[1] Brenchley JM, Price DA, Schacker TW, Microbial translocation is a cause of systemic immune activation in chronic HIV infection. Nat Med. 2006;12(12):1365-1371. doi:10.1038/nm1511 17115046

[2] Purohit V, Bode JC, Bode C, Alcohol, intestinal bacterial growth, intestinal permeability to endotoxin, and medical consequences: summary of a symposium. Alcohol. 2008;42(5):349-361. doi:10.1016/j.alcohol.2008.03.131 18504085 PMC2614138

[3] Deeks SG HIV infection, inflammation, immunosenescence, and aging. Annu Rev Med. 2011;62:141-155. doi:10.1146/annurev-med-042909-093756 21090961 PMC3759035

[4] Galvan FH, Bing EG, Fleishman JA, The prevalence of alcohol consumption and heavy drinking among people with HIV in the United States: results from the HIV Cost and Services Utilization Study. J Stud Alcohol. 2002;63(2):179-186. doi:10.15288/jsa.2002.63.179 12033694

[5] Samet JH, Walley AY Interventions targeting HIV-infected risky drinkers: drops in the bottle. Alcohol Res Health. 2010;33(3):267-279. 23584068 PMC3860515

[6] Bertholet N, Daeppen JB, Wietlisbach V, Fleming M, Burnand B Reduction of alcohol consumption by brief alcohol intervention in primary care: systematic review and meta-analysis. Arch Intern Med. 2005;165(9):986-995. doi:10.1001/archinte.165.9.986 15883236

[7] Bao B, Prasad AS, Beck FW, Zinc decreases C-reactive protein, lipid peroxidation, and inflammatory cytokines in elderly subjects: a potential implication of zinc as an atheroprotective agent. Am J Clin Nutr. 2010;91(6):1634-1641. doi:10.3945/ajcn.2009.28836 20427734 PMC2869512

[8] Baum MK, Shor-Posner G, Campa A Zinc status in human immunodeficiency virus infection. J Nutr. 2000;130(5S)(suppl):1421S-1423S. doi:10.1093/jn/130.5.1421S 10801954

[9] Visser ME, Durao S, Sinclair D, Irlam JH, Siegfried N Micronutrient supplementation in adults with HIV infection. Cochrane Database Syst Rev. 2017;5:CD003650. doi:10.1002/14651858.CD003650.pub4 28518221 PMC5458097

[10] Office of Dietary Supplements/National Institutes of Health Dietary supplement fact sheet: zinc. Accessed December 13, 2010. https://ods.od.nih.gov/factsheets/Zinc-QuickFacts/

[11] Lambert JC, Zhou Z, Wang L, Song Z, McClain CJ, Kang YJ Preservation of intestinal structural integrity by zinc is independent of metallothionein in alcohol-intoxicated mice. Am J Pathol. 2004;164(6):1959-1966. doi:10.1016/S0002-9440(10)63756-X 15161632 PMC1615750

[12] Lambert JC, Zhou Z, Wang L, Song Z, McClain CJ, Kang YJ Prevention of alterations in intestinal permeability is involved in zinc inhibition of acute ethanol-induced liver damage in mice. J Pharmacol Exp Ther. 2003;305(3):880-886. doi:10.1124/jpet.102.047852 12626662

[13] Baum MK, Lai S, Sales S, Page JB, Campa A Randomized, controlled clinical trial of zinc supplementation to prevent immunological failure in HIV-infected adults. Clin Infect Dis. 2010;50(12):1653-1660. doi:10.1086/652864 20455705 PMC2874106

[14] Dirajlal-Fargo S, Yu J, Kulkarni M, Brief report: zinc supplementation and inflammation in treated HIV. J Acquir Immune Defic Syndr. 2019;82(3):275-280. doi:10.1097/QAI.0000000000002129 31609926 PMC6812547

[15] Gnatienko N, Freiberg MS, Blokhina E, Design of a randomized controlled trial of zinc supplementation to improve markers of mortality and HIV disease progression in HIV-positive drinkers in St. Petersburg, Russia. HIV Clin Trials. 2018;19(3):101-111. doi:10.1080/15284336.2018.1459344 29663871 PMC5957784

[16] Justice AC, McGinnis KA, Skanderson M, ; VACS Project Team Towards a combined prognostic index for survival in HIV infection: the role of ‘non-HIV’ biomarkers. HIV Med. 2010;11(2):143-151. doi:10.1111/j.1468-1293.2009.00757.x 19751364 PMC3077949

[17] Ridker PM, Buring JE, Rifai N, Cook NR Development and validation of improved algorithms for the assessment of global cardiovascular risk in women: the Reynolds Risk Score. JAMA. 2007;297(6):611-619. doi:10.1001/jama.297.6.611 17299196

[18] Justice AC, Freiberg MS, Tracy R, ; VACS Project Team Does an index composed of clinical data reflect effects of inflammation, coagulation, and monocyte activation on mortality among those aging with HIV? Clin Infect Dis. 2012;54(7):984-994. doi:10.1093/cid/cir989 22337823 PMC3297653

[19] National Institute on Alcohol Abuse and Alcoholism Helping Patients Who Drink Too Much: A Clinician’s Guide—Updated. National Institutes of Health; 2007.

[20] Fawzi WW, Villamor E, Msamanga GI, Trial of zinc supplements in relation to pregnancy outcomes, hematologic indicators, and T cell counts among HIV-1-infected women in Tanzania. Am J Clin Nutr. 2005;81(1):161-167. doi:10.1093/ajcn/81.1.161 15640476

[21] Kazis LE, Miller DR, Clark J, Health-related quality of life in patients served by the Department of Veterans Affairs: results from the Veterans Health Study. Arch Intern Med. 1998;158(6):626-632. doi:10.1001/archinte.158.6.626 9521227

[22] Heatherton TF, Kozlowski LT, Frecker RC, Fagerström KO The Fagerström Test for Nicotine Dependence: a revision of the Fagerström Tolerance Questionnaire. Br J Addict. 1991;86(9):1119-1127. doi:10.1111/j.1360-0443.1991.tb01879.x 1932883

[23] Chesney MA, Ickovics JR, Chambers DB, ; Patient Care Committee & Adherence Working Group of the Outcomes Committee of the Adult AIDS Clinical Trials Group (AACTG) Self-reported adherence to antiretroviral medications among participants in HIV clinical trials: the AACTG adherence instruments. AIDS Care. 2000;12(3):255-266. doi:10.1080/09540120050042891 10928201

[24] Sobell LC, Sobell MB Alcohol Timeline Followback (TLFB) Users’ Manual. Addiction Research Foundation; 1995.

[25] Herron AJ, Mariani JJ, Pavlicova M, Assessment of riboflavin as a tracer substance: comparison of a qualitative to a quantitative method of riboflavin measurement. Drug Alcohol Depend. 2013;128(1-2):77-82. doi:10.1016/j.drugalcdep.2012.08.007 22921475 PMC3556739

[26] Olechnowicz J, Tinkov A, Skalny A, Suliburska J Zinc status is associated with inflammation, oxidative stress, lipid, and glucose metabolism. J Physiol Sci. 2018;68(1):19-31. doi:10.1007/s12576-017-0571-7 28965330 PMC5754376

