# Supplementary material for: Effect of Zinc Supplementation vs Placebo on Mortality Risk and HIV Disease Progression Among HIV-Positive Adults With Heavy Alcohol Use: A Randomized Clinical Trial
Source: JAMA Netw Open. 2020 May 8;3(5):e204330. doi: 10.1001/jamanetworkopen.2020.4330 (PMC7210486; doi:10.1001/jamanetworkopen.2020.4330)
Supplement: Supplement 1. — Trial Protocol [file jamanetwopen-3-e204330-s001.pdf]

STUDY PROTOCOL

Zinc for INflammation and Chronic disease in HIV (ZINC HIV)

Principal Investigators:

Jeffrey H. Samet, MD, MA, MPH – Boston Medical Center (contact)

Matthew S. Freiberg, MD, MSc - Vanderbilt University Medical Center

NIAAA Award Number: U01AA021989

Clinicaltrials.gov Registration: NCT01934803

Boston University Medical Campus IRB Protocol Number: H-31901

Enrollment Dates: October 2013 – June 2015

Protocol Version: FINAL

TABLE OF CONTENTS

1. INTRODUCTION #5

1.1 SUMMARY …. **#5**

1.2 SIGNIFICANCE …**#5**

2. OVERVIEW OF STUDY DESIGN #5

2.1 STUDY AIMS AND OUTCOMES … **#5**

2.2 STUDY HYPOTHESES …**#6**

2.3 STUDY DESIGN … **#6**

2.4 STUDY SITE …**#7**

2.5 INCLUSION CRITERIA … **#7**

2.6 EXCLUSION CRITERIA AT STUDY ENTRY …**#8**

2.7 RECRUITMENT GOALS …**#8**

2.7.A. SAMPLE SIZE CALCULATION AND POWER …**#8**

3. INTERVENTION #8

3.1 INTERVENTION OVERVIEW …**#9**

3.2 RANDOMIZATION …**#9**

3.3 INTERVENTION … **#9**

3.3.A. CONTROL … **#10**

3.4 MEDICATION CONSIDERATIONS …**#10**

3.4.A. SYMPTOM MONITORING …**#10**

3.4.B. ADHERENCE …**#11**

3.4.C. MEDICATION DISBURSEMENT …**#12**

3.4.D. LOST OR STOLEN STUDY MEDICATION …**#12**

3.4.E. DISCONTINUATION OF STUDY MEDICATION …**#12**

3.5 SCHEDULE OF DATA COLLECTION …**#13**

3.5.A. VISIT WINDOWS ………… #13
3.6 DATA SOURCES ………… #14
3.6.A QUESTIONNAIRES ………… #14
3.6.B. BLOOD ………… #14
3.6.C. URINE ………… #15
3.6.D. LIVER STATUS ………… #16
3.6. E. DRIED BLOOD SPOTS ………… #16

4. STUDY PROCEDURES #16

4.1 RECRUITMENT ………… #16
4.2 SCREENING ………… #17
4.3 INFORMED CONSENT ………… #18
4.4 VISIT FLOW ………… #18
4.5 QUALITY ASSURANCE ………… #19
4.6 COMPENSATION ………… #20
4.7 RETENTION ………… #20

5. ASSESSMENTS #22

5.1 BASELINE ASSESSMENT ………… #22
5.2 FOLLOW UP ASSESSMENTS ………… #22
5.2.A. MEDICATION VISITS ASSESSMENTS ………… #22

6. PARTICIPANT SAFETY #23

6.1 SPECIFICATION OF SAFETY PARAMETERS ………… #24
6.2 THE METHODS AND TIME FOR ASSESSING, RECORDING, AND ANALYZING SAFETY PARAMETERS ………… #25
6.3 PROCEDURES FOR ELICITING REPORTS OF AND FOR RECORDING AND REPORTING ADVERSE EVENT AND INTERCURRENT ILLNESSES ………… #27
6.4 THE TYPE AND DURATION OF THE FOLLOW-UP OF SUBJECTS AFTER ADVERSE EVENTS ………… #28
6.5 UNBLINDING PROTOCOL ………… #28

6.6 **DATA SAFETY AND MONITORING BOARD**……………………………………………….….…#**29**

7. DATA MANAGEMENT **#30**

7.1 **DATA COLLECTION**…………………………………………………………………........#**30**

7.2 **QUALITY CONTROL PROCESS**... **#30**

7.3 **DATA SECURITY AND CONFIDENTIALITY**……………………………………………......#**30**

7.4 **WEB SYSTEMS**…………………………………………………………………………....#**30**

8. STATISTICAL ANALYSIS **#33**

8.1 **PRIMARY ANALYSES**…………………………………………………….……......#**33**

8.2 **ADDITIONAL EXPLORATORY ANALYSES**…..………………………………………….…....#**34**

9. STAFF TRAINING **#35**

10. STUDY CONTACTS **#35**

1. INTRODUCTION

1.1 SUMMARY

The combination of heavy alcohol consumption and HIV infection is associated with increased mortality, HIV disease progression, acute myocardial infarction (AMI) and a proinflammatory state characterized by increased biomarker levels of inflammation. Heavy alcohol use and HIV infection are both causes of microbial translocation, the process by which bacterial products from the gastrointestinal (GI) tract leak across the GI membrane to the portal circulation. Microbial translocation causes immune activation leading to end organ damage. Alcohol can cause microbial translocation via zinc deficiency. Zinc deficiency is common among HIV+ heavy drinkers and linked to high mortality rates. Zinc supplementation is affordable, available, does not interfere with ART, and has minimal adverse drug reactions. In animal models zinc reduces ethanol associated microbial translocation. In human studies zinc slows HIV disease progression and reduces levels of inflammatory biomarkers which are strongly linked to mortality. Given zinc's potential efficacy we will conduct Zinc for INflammation and Chronic disease in HIV (ZINC HIV), a double-blinded randomized controlled trial to assess the efficacy of zinc supplementation vs. placebo among 250 HIV+ Russians, who are ART-naïve at enrollment and have a recent history of heavy drinking. Our specific aims will test the efficacy of zinc supplementation, compared to placebo to (1) improve markers of mortality as measured by the VACS index; (2) slow HIV disease progression as measured by CD4 cell count; (3) improve markers of AMI risk as measured by the Reynolds risk score; and (4) lower levels of microbial translocation and inflammation as measured by serum biomarkers. We hypothesize that as compared with placebo, patients receiving zinc supplementation will have significantly lower AMI and mortality risk as measured by the VACS index and Reynolds risk scores; higher CD4 cell counts; lower levels of biomarkers for microbial translocation and inflammation. Importantly, if our hypotheses are true, zinc supplementation could ultimately become a standard adjunctive therapy complementing alcohol interventions among HIV+ persons even in resource limited environments.

1.2 SIGNIFICANCE

The combination of heavy alcohol consumption and HIV infection results in serious health problems and an increased risk of death. Although the mechanism is not clear, inflammation appears to play an important role. Zinc supplementation has anti-inflammatory properties. This study is designed to see if giving zinc supplementation to HIV-positive people who are heavy drinkers reduces the risk of serious health problems and death.

2. OVERVIEW OF STUDY DESIGN

2.1 STUDY AIMS AND OUTCOMES

ZINC aims to test the efficacy of zinc supplementation, compared to placebo, to:

1. Improve markers of mortality as measured by the change in VACS index between baseline and 18 months—Primary outcome

2. Slow HIV disease progression as measured by change in CD4 cell count between baseline and 18 months—Secondary outcome

3. Improve markers of AMI risk as measured by the Reynolds risk score at 18 months—Secondary outcome

4. Lower biomarker levels of microbial translocation and inflammation at 18 months—Secondary outcome

2.2 STUDY HYPOTHESES

We hypothesize that as compared with placebo, participants receiving zinc supplementation will have significantly:

Hypothesis 1- Smaller change in VACS (Primary);

Hypothesis 2- Greater change in CD4 cell counts (Secondary);

Hypothesis 3- Lower Reynolds risk score (Secondary);

Hypothesis 4- Lower biomarker levels of microbial translocation and inflammation (Secondary).

2.3 STUDY DESIGN

ZINC is a double-blinded randomized placebo-controlled trial of zinc supplementation (Zinc for INflammation and Chronic disease in HIV [ZINC]) among HIV-positive heavy drinkers in Russia to evaluate the efficacy of zinc to 1) improve markers of mortality, as measured by the VACS index; 2) slow HIV disease progression, as measured by CD4 cell count; 3) improve markers of coronary heart disease (CHD) risk, as measured by the Reynolds risk score and; 4) decrease microbial translocation and inflammation, as measured by serum biomarkers. Participants will receive study medication over 18 months, with study visits occurring at 6, 12, and 18 months post enrollment, and shorter medication adherence visits at 6 weeks, 12 weeks, 9 months, and 15 months. ZINC RCT is nested within the Russia ARCH cohort of the Uganda, Russia, Boston Alcohol Network for Alcohol Research Collaboration on HIV/AIDS (URBAN ARCH) Consortium, which aims to understand how alcohol use impacts people affected by HIV and develop interventions to reduce alcohol use and alcohol and HIV-related

consequences in this population.

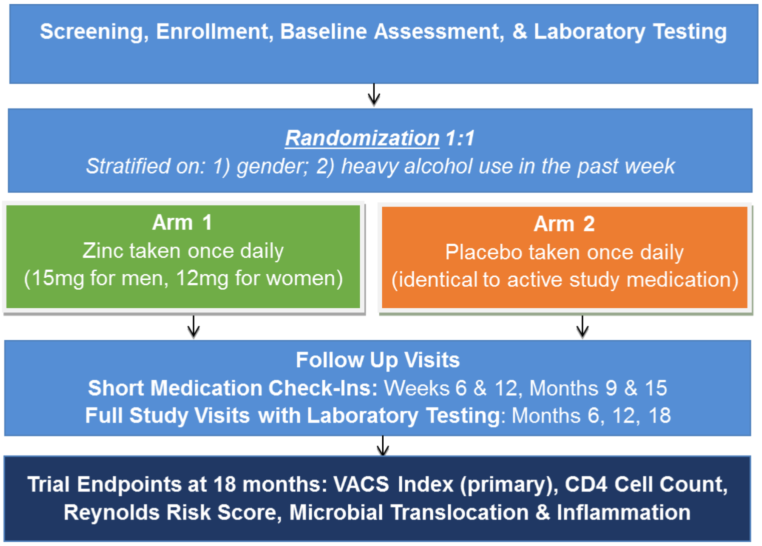

2.4 STUDY SITE

Recruitment, enrollment, and all study visits will take place at the Laboratory of Clinical Pharmacology of Addictions at the First St. Petersburg Pavlov State Medical University (PSMU) in St. Petersburg, Russia. PSMU is the major educational, scientific, and clinical medical institution for northwestern Russia. Blood specimens will be processed and analyzed at ImmunoBioService (IBS) and Pasteur Laboratories.

2.5 INCLUSION CRITERIA

To be eligible to participate in the trial, participants must meet the following inclusion criteria:

1. 18-70 years old

2. Documented HIV-positive

3. Documented ART-naïve status

4. Heavy alcohol consumption (i.e., NIAAA risky drinking criteria: > 4 standard drinks in a day [or > 14 standard drinks/week] for men and > 3/day [or 7/week] for women) in the past 30 days

5. Ability to provide contact information for two contacts to assist with follow-up

6. Stable address within St. Petersburg or districts within 100 kilometers of St. Petersburg

7. Possession of a home or mobile phone.

2.6 EXCLUSION CRITERIA AT STUDY ENTRY

1. Not fluent in Russian
2. Cognitive impairment resulting in inability to provide informed consent based on research assessor (RA) assessment
3. Pregnancy, planning to become pregnant, or breast feeding

2.7 RECRUITMENT GOALS

The study aims to recruit 250 participants.

Note: With permission from NIAAA, the study exceeded its target enrollment of 250 to enroll 254 heavy drinkers to account for enrollment of 4 participants who were subsequently discovered to be HIV-negative.

2.7.A. SAMPLE SIZE CALCULATION AND POWER

Power was calculated for the overall primary study endpoint (change in VACS index score). Power calculations assumed a two-sided hypothesis test, with a significance level of 0.05. It was expected that 250 participants would be enrolled into the study. We anticipated 20% loss to follow-up due to death and participant withdrawal. Based on the VACS study, the standard deviation of the change in VACS index score from initiation of ART to after one year was 25. We expect the standard deviation will be similar in this study. Given these assumptions and with 200 evaluable participants (assuming 20% loss to follow-up, and 100 participants in each arm), the study is anticipated to have 80% power to detect a difference between the placebo and zinc groups in their mean changes in VACS index score over the 18-month period as small as 10 (e.g. 20 vs. 10 for the placebo and zinc groups, respectively) using a two-sided t-test.

Aim 2: To test the efficacy of zinc supplementation compared to placebo to slow HIV disease progression as measured by CD4 cell count. We anticipate analyzing log transformed values of change in CD4 cell count due to possible skewness in the data. Group differences will be back transformed from the natural log scale and therefore the parameter of interest is the ratio of the mean changes in CD4 cell count from baseline to 18 months for the zinc group relative to the placebo group, where change is defined as CD4 count at baseline - CD4 count at 18 months. The estimated coefficient of variation for the change in CD4 cell count from baseline to 18 months (baseline - 18 months) is 0.20 cells/μL. Given these assumptions, with 200 evaluable participants (100 in the zinc and placebo arms), the study will have 96.3% power to detect a ratio of 0.90 (zinc:placebo) between the mean changes of CD4 counts for the zinc and placebo groups using a two-sided t-test.

3. INTERVENTION

3.1 INTERVENTION OVERVIEW

ZINC is a double-blinded randomized control trial to assess the efficacy of zinc supplementation vs. placebo among HIV-positive Russians, who are ART-naïve at enrollment with a recent history of heavy drinking. Participants will be randomly assigned to receive either zinc supplementation or placebo to be taken orally daily over 18 months.

3.2 RANDOMIZATION

ZINC randomization will be assigned in a 1:1 ratio to zinc or placebo utilizing block randomization where stratification will be based upon gender and heavy alcohol consumption during the past week to ensure balance. The randomization, stratification and assignment of participants to the two treatment groups will be conducted and monitored by the URBAN ARCH Biostatistics and Data Management (BDM) core. The software package SAS will be used to generate randomization lists to assign participants as they are enrolled into the clinical trial. The study pharmacist will receive the list of randomization IDs with group assignment and will provide packaged boxes of study medication to the study team.

Following completion of the baseline assessment, the RA will be directed to the electronic randomization screen, which will provide a checklist of all components that need to be completed prior to randomization. Randomization will not be able to proceed until all outstanding questions are resolved (e.g., contact information and 7-day alcohol use entered into the electronic system). Once submitted, the randomization page will automatically assign the participant to a randomization ID. The RA will then retrieve the box of study medication labeled with the correct randomization ID and label the box with the participant study ID, thus linking the two numbers. As a double-blinded study, ZINC participants, investigators, RAs and the study nurse will be unaware of participant group assignment.

3.3 INTERVENTION

Participants randomly assigned to the intervention (zinc) group will receive study medication at all but the final visit. Of the eight study visits, four include longer assessments with lab work and four are shorter medication adherence checks.

Zinc capsules will be compounded using pharmacy-grade zinc gluconate and Riboflavin (adherence measure) at Bios Pharmaceuticals in St. Petersburg. Zinc capsules for men and women will contain 15 and 12 mg of zinc gluconate, respectively, as this dose had been shown to be effective in previous trials and has minimal risk of adverse events. The capsules will be provided in bottles, each containing a 28-day supply of medication, labeled differently for males and females.

Participants will be instructed to take one pill daily by mouth with a full glass of water, at the same time they take any other medications. If participants are not taking any other medications, they will be told to take the study drug at the same time each day. Participants will be instructed to not eat food or drink beverages containing caffeine 1-2 hours before or after taking the medication and to never take more than one pill per day. The latter is due to its potential interaction with zinc absorption.

Individuals using zinc supplements could experience adverse effects if they take more zinc than provided. These adverse effects could include nausea, vomiting, diarrhea, and abdominal pain. Normally, these adverse effects are seen in people who regularly consume at least 40mg-1000mg/day. In order to help prevent these adverse effects, participants will be instructed not to take more zinc supplements than what was provided by the study. Study research staff will also monitor participants for signs of zinc overdose. These adverse effects are not expected to be likely.

Zinc may decrease the body's absorption of two kinds of antibiotics, quinolones and tetracyclines, which include: Ciprofloxacin (Cipro), Levofloxacin (Levaquin), Ofloxacin (Floxin), Moxifloxacin (Avelox), Norfloxacin (Noroxin), Gatifloxacin (Tequin), Tetracycline, Minocycline (Minocin), Demeclocycline (Declomycin), Cinoxacin (Cinobac), Enoxacin (Penetrex), Gemifloxacin (Factive), Grepafloxacin (Raxar), Sparfloxacin (Zagam). Participants will be advised to discontinue taking study medications while they are on antibiotics and to consult a physician for further guidance and information.

In accordance with FDA guidelines, the study did not require an Investigational New Drug (IND) application, as it was conducted outside of the United States.

3.3.A CONTROL

Participants randomized to the control group will receive all study procedures and instructions identical to the intervention group. Participants will receive a sucrose placebo identical to the zinc medication in appearance and taste. Riboflavin will be added to both active and placebo medication as a biologic adherence measure. Sucrose, for the placebo and Riboflavin, for the biologic adherence measure, will be obtained from the same pharmacy as the study medication for the intervention group.

3.4 MEDICATION CONSIDERATIONS

3.4.A SYMPTOM MONITORING

To minimize medication risks, participants will be monitored for adverse effects at each study visit. Participant symptoms will be assessed at baseline and any chronic conditions or symptoms that existed prior to introduction of study medication will be documented using an electronic Baseline Event form. During each subsequent study visit, the RA will ask the participant how they feel and review the list of symptoms of concern, beginning with any symptoms recorded at the previous visit and including the four

most frequent side effects of zinc: abdominal pain, diarrhea, nausea, and vomiting. The RA will ask about any new symptoms experienced by the participant since the last study visit. Any event that meets the criteria for an adverse event (AE), serious adverse event (SAE), or unanticipated problem will be recorded using paper and electronic AE and/or SAE forms. The site will receive the results of all blood work performed on study participants. Any abnormal lab results that are deemed clinically significant by the clinical team will be recorded as an AE and/or SAE and the participant will be referred to their local medical provider. All baseline events, AE and SAE forms will be reviewed by the US and Russian teams. All events will be presented to and reviewed by the Data and Safety Monitoring Board (DSMB).

Participants will also be alerted of possible interactions between zinc and certain antibiotics and instructed to discontinue the study drug and contact the study team should they initiate a course of antibiotic medication.

Participants will be encouraged to contact study personnel or another health care provider if they experience any adverse effects. Furthermore, every two months a report will be generated of participants who report taking more than on pill per day, or for those whose pill count adherence was greater than 100% at their previous study visit. The report will be reviewed by the US Project Manager and the Russian study team. Any questions or concerns will be brought forth to the study PIs.

3.4.B. ADHERENCE

Medication adherence will be assessed at each study visit using direct (Riboflavin) and indirect (pill counts and self-report) measures.

Direct Adherence Measures

Riboflavin (50 mg), a vitamin yielding a change in urine color, will be added to both active and placebo capsules. Participants will be informed that the color change is harmless. At this dose, Riboflavin is expected to remain in the system at detectable levels for up to 24 hours. At each study visit post-baseline (while taking study medication), participants will be asked to provide a urine sample, which will be visually inspected for the presence or absence of Riboflavin in a room with low ambient light, using ultraviolet (UV) light at the long wave setting (33 mm).

Indirect Adherence Measures

Pill Counts

Participants will be instructed to bring any unused medication to each study visit post-baseline. The RA will count and record the number of remaining pills.

Self-Report

Medication adherence will also be measured through self-report using the modified Adult AIDS Clinical Trial Group (AACTG) ART adherence questions. At each post-baseline study visit participants will be

asked to draw a line on a paper ruler, numbered from 0-100, to indicate the number best representative of how much of the study medication they have taken in the past 6 weeks. Participants will also be asked questions about the longest period of time in which they consistently took the study medication, if they stopped taking the medication, and if they took more than one pill of study medication on any day in the past 6 weeks.

Adherence Aids

During each study visit (for the exception of the 18-month visit) medication instructions will be reviewed and strategies for adherence will be discussed with each participant. Adherence plans will be individually tailored to each participant, depending on their reason for non-adherence. To further increase medication adherence, an automated text message will be sent twice per week for the first 6 weeks, reminding participants to take their study medication. At the 6-week appointment, the assessor will ask the participant whether the text messages are helpful and whether to continue, increase or decrease the frequency of the reminders. Participants will be able to reduce the frequency or opt out of text message reminders entirely at any time throughout the study.

3.4.C. MEDICATION DISBURSEMENT

Medication inserts will be provided to participants at baseline.

Participants will receive an extra 6-week of supply of study medication to accommodate instances of lost medication or missed visits. At subsequent study visits, the participant will be asked to bring in any unused medications. The assessor will count the number of remaining pills and redistribute them to the participant.

The pharmacist will deliver, at one time, six months-worth of study medications to the assessors at Pavlov State Medical University.

3.4.D. LOST OR STOLEN STUDY MEDICATION

If participants report lost or stolen medication, they will be provided with extra study medication. In the event that a participant reports losing medication more than once, the study team will be alerted and the case discussed to determine a plan of action.

3.4.E. DISCONTINUATION OF STUDY MEDICATION

Those who discontinue medication will be followed and analyzed by intention to treat.

Participants found to be pregnant during the study will have their study medication discontinued, but will still be followed-up for the duration of the study. Participants who report pregnancy outside of study visits will be instructed to immediately discontinue their study medication and asked to come in for a confirmatory urine pregnancy test.

3.5 Schedule of Data collection

3.5.A. Visit windows

1. Screener B/Baseline Visit:
   - Window open: Date screened (A)
   - Window close: 30 days after being screened (A)
   - Window length: 30 days

Following 30 days, screeners A and B (see 4.2 Screening) will be administered again if individual is interested in participating.

2. 6 Week Visit
   - Window open: 5 weeks post baseline
   - Window close: 9 weeks post baseline
   - Window length: 4 weeks (1 month)

3. 12 Week Visit (3 months)
   - Window open: 9 weeks post baseline
   - Window close: 16 weeks (4 months) post baseline
   - Window length: 7 weeks

4. 6 Month Visit
   - Window open: 4 months post baseline
   - Window close: 8 months post baseline
   - Window length: 4 months

5. 9 Month Visit
   - Window open: 8 months post baseline
   - Window close: 11 months post baseline
   - Window length: 3 months

6. 12 Month Visit

Window open: 11 months post baseline

Window close: 14 months post baseline

Window length: 3 months

7. 15 Month Visit

Window open: 14 months post baseline

Window close: 17 months post baseline

Window length: 3 months

8. 18 Month Follow Up

Window open: 17 months post baseline

Window close: 21 months post baseline

Window length: 4 months

3.6 DATA SOURCES

3.6.A QUESTIONNAIRES

Participants will be assessed at baseline, 6-, 12- and 18-months post enrollment, along with shorter medication visits at 6-weeks, 12-weeks, 9-months and 15-months. All study assessments will take place at First St. Petersburg Pavlov State Medical University. On occasions when a study participant is unable to come to the study site for a face-to-face interview and is danger of falling out of their assessment window, RAs will conduct study assessments over the telephone. In addition to study questionnaires, at each study visit RAs will measure and record participants' height, weight, and blood pressure.

3.6.B. BLOOD

This study requires the collection of 22 mL of blood at baseline, 6-, 12-, and 18-month study visits. Blood will be tested for hemoglobin, platelets, CD4 count, HCV antibody and qualitative viral load (baseline and 18-months only), HIV viral load, high sensitivity C-reactive protein (HS CRP), total and HDL cholesterol (baseline and 18-months only), eGFR (creatinine), AST/ALT, and zinc levels (baseline and 18-months only). Zinc level testing will be performed in batches at ImmunoBioService laboratory. The remainder of the testing will be conducted at the Pasteur Laboratory.

Plasma samples will be stored for biomarker testing. Dried blood spot cards will be spotted using 60μL of blood per spot (or 300μL per card) and saved to be used for phosphatidylethanol (PEth) alcohol

biomarker testing. The incorporation of PEth will provide complement to self-report for alcohol consumption.

Blood will be collected prior to the assessment at baseline, 6-, 12-, and 18-month study visits. For a blood draw to be considered successful, one EDTA (8 mL) and one SST (3 mL) tube both must be at least ¾ full. If the draw is successful, participants will be provided with full compensation and continue with the standard ZINC visit procedures. If the blood draw is unsuccessful at the participant's baseline visit, the participant will receive partial (1/3) compensation. The participant has 30 days after completion of the Screener to complete the blood draw. The participant will be disenrolled after three unsuccessful blood draw attempts or if more than 30 days have passed. The participant will be provided with full compensation at the third or final attempt. If the blood draw is unsuccessful at a follow-up study visit, the participant will be given partial (1/2) compensation for completion of the assessment. The participant will have 7 days to complete the second blood draw attempt. If this attempt is unsuccessful, the third attempt will be done at the next study visit (medication only visit). The participant will be provided with full compensation at the third attempt. For blood draw attempts that occur outside of the 7-day window, sections on medications and past 24-hour activities will be reassessed.

The following algorithm will be followed at the 18-month study visit for retesting HCV Ab/VL:

- If the participant is AB negative at baseline, they will be tested for Ab at 18-months, and if positive tested for VL. The rationale for this is that if the participant was found to be newly Ab positive at 18-months this would signal possible new HCV exposure requiring confirmation. If the VL is positive, this confirms new infection.
- If the participant is Ab positive and VL negative at baseline, they would only be tested for VL at 18-months. HCV Ab is not protective and therefore the individual remains at risk for re-infection if exposure, thus, necessitating re-testing VL at 18-months. While the additional nuance that there are a small number of patients who have a low fluctuating VL after initial infection, which may be intermittently negative before transitioning to chronic infection with high VL exists, this is beyond is the scope of the study. For this study, we presumed that VL negative at baseline and VL positive at 18-months indicated new exposure and infection.
- Participants who are Ab and VL positive at baseline will not be re-tested at 18-months.

All participants will be offered pre-and post-counseling for HCV testing for new diagnoses.

Please see the study laboratory protocol for additional detailed information regarding phlebotomy and sample processing procedures.

3.6.C. URINE

A pregnancy test will be administered by trained clinical research staff at screening to determine eligibility and at each study visit.

Urine will also be used to measure adherence to the study medications via the added Riboflavin, as described in section on Adherence.

3.6.D. Liver status

As many of the biomarkers used to assess inflammation are synthesized in the liver, an inability to account for liver health makes interpretation of the biomarkers of inflammation (e.g., CRP) and their association with zinc and HIV disease progression, as well as, AMI and mortality risks difficult. Therefore to minimize confounding due to liver disease, two baseline measures of liver health (fibroscan and FIB-4 score) will be obtained. Fibroscan is a non-invasive ultrasonic imaging technique used as an alternative to liver biopsy. FIB-4 score is an inexpensive and accurate biomarker of liver fibrosis in HIV- and HCV-positive patients and those with alcohol disorders. The study team will calculate FIB-4 scores using baseline laboratory values for all study participants. Through consultation with a liver expert, the study team created an algorithm to place participants in one of three categories based on their FIB-4 values: high, low, and unclear possibility of liver cirrhosis. Participants that fall into the “unclear” category (FIB-4 values ranging from 1.4-3.25) will receive a fibroscan at their next study visit to determine their liver disease status. Fibroscans will be conducted at the First St. Petersburg Pavlov State Medical University at the participant’s 6-week assessment.

3.6.E. Dried blood spots

Dried blood spots (DBS) will be collected at each study visit for phosphatidylethanol (PEth) testing, which will be conducted at the United States Drug Testing Laboratories, Inc. (USDTL).

4. STUDY PROCEDURES

4.1 RECRUITMENT

Participants will be recruited from the following sources:

- Russia ARCH cohort (U01AA020780)
- Notification of patients at clinical and non-clinical sites of care affiliated with Pavlov State Medical University (PSMU) through distribution of study flyers. These sites include Botkin Infectious Disease Hospital, St. Petersburg AIDS Center, City Addiction Hospital and local non-governmental organizations (NGOs) serving HIV-positive persons. Staff at these sites will be informed about the study and asked to refer interested and potentially eligible persons to our recruitment site (Pavlov Medical University) for phone or in-person screening by our study research team.
- Participant database of our laboratory at Pavlov Medical University
- Enrolled participants (i.e. snowball recruitment). Enrolled participants will be given information sheets to distribute to potential participants who might be interested and qualified. Interested

potential participants will be responsible for directly contacting study staff listed on the information sheet to be screened for eligibility.

4.2 SCREENING

Existing Russia ARCH participants will be offered an opportunity to be screened for ZINC during one of their ARCH study-related contacts (phone or in-person); in addition, some individuals will be screened for Russia ARCH and ZINC simultaneously.

If an interested participant reaches out to the study team (or is reached by study team) via phone or in-person (Pavlov University), the RA will administer a verbal consent for the screening process, which will include a brief description of the study. Following receipt of verbal consent the RA will screen the participant to confirm eligibility. All responses to the screener will be entered directly into an electronic data capture system by the RA. Screening for the study will occur in two steps: A (verbal) and B (with documentation).

To ensure that double enrollment does not occur, prior to screening, the assessor will search in the electronic system for the last name of the potential participant. If the name does not already exist in the system, the assessor will proceed with screening the participant. If the name exists in the system, the assessor will compare the first name, age and gender of the potential participant with that of the participant with the same last name to determine if the individual has already been enrolled (this will be done very confidentially, ensuring that the potential participant will not be able to ascertain that there is someone with the same last name already in the study, to protect the confidentiality of the enrolled participant). If all of these characteristics match, but the individual says they never enrolled before, the assessor will contact the Russian study supervisor prior to enrollment to determine whether or not this person was previously enrolled.

The RA will administer ZINC screener A to confirm the participant's age; ART-status; that the participant is not currently pregnant or breastfeeding; that the participant has a home or mobile telephone; and lives within 100 kilometers of St. Petersburg. The RA will also ask about the participant's quantity and frequency of drinking in the past 30 days.

Once a participant is confirmed to meet study entry criteria (screening A), they will be scheduled for their first study visit (screening B and baseline). If the participant was already enrolled in ARCH, but new to ZINC, their baseline visit will be scheduled to coincide with an ARCH follow-up visit. If the participant is new to both ARCH and ZINC, an RA will call to remind the participant of their visit both one week and 24 hours before their scheduled appointment (if time allows).

When the participant arrives at Pavlov University for their baseline visit, the RA will re-screen the participant to assess ART use, pregnancy and breastfeeding status, and confirm HIV and ART status (screening B). As part of screening B, participants will be required to provide documentation of HIV and ART-naïve status. This documentation will take form of letters from a medical provider, laboratory results and excerpts from medical histories.

A 30-day window is allowed between screener A and screener B/baseline. Participants who are unable to return to the laboratory within 30 days (either due to scheduling or inability to obtain documentation) will re-initiate the screening process with screening consent and screener A.

Participants who are found ineligible at either stage (screener A or B) will be thanked for their time and will receive a listing of local addiction and HIV care resources. Data collected on participants who "screen out" will be kept in order to have an accurate record of the rate of enrollment among participants screened for participation, and to be able to identify reasons why potential participants are ineligible. The data will not contain identifying information.

4.3 INFORMED CONSENT

After eligibility and interest in enrollment is determined, a research assessor will administer and documented the informed consent of the participant. If the potential participant needs time to consider participating, the RA will provide his/her contact information to the participant. If the RA has not heard from the participant 3 days after the initial screening, s/he will call the participant back to ask about participating. If the participant is still not ready to accept or refuse participation, s/he will be able to call the RA at anytime but will need to be re-screened. The study will be explained to eligible participants and research assessors will answer any questions the participants have, including risks, benefits and alternatives (including non-participation) to participation, and will provide written materials describing the study. The written informed consent (in Russian), including the risks, benefits and alternatives, will be signed by the participant and the research assessor. As part of the informed consent process, it will be made explicit to the participants that their involvement in the study will not constitute medical treatment and that they would not receive any medical care (HIV or addiction) as part of the study. A handout will be provided with information on addiction and HIV treatment services to participants at the baseline visit. A copy of the informed consent will be provided to the participant and a copy will be maintained by the research team. Potential participants will be informed that refusal to participate would not affect their medical care at PSMU in any way and they will be informed of their right to drop out of the study at any time.

Once the participant signs the informed consent form, the RA will finalize and complete the Consent and Enrollment Form electronically.

4.4 VISIT FLOW

Baseline:

Participants will be asked to complete a baseline assessment immediately after signing the informed consent. The baseline assessment will include a face-to-face- questionnaire and collection of blood from participants.

The baseline assessment will take between one and two hours to complete.

Follow Up Assessment:

The assessor will administer the Symptom Monitoring Form. Following this, a urine sample will be collected. The urine sample will be tested for pregnancy and assessed for color change using a UV light. Results will be recorded in the ZINC Medication Adherence Form. The assessor will conduct the study assessment and send the participant for a blood draw. The face-to-face interview for the 6, 12, and 18-month follow up assessment will follow the same protocol as the baseline assessment in that there will be an interviewer-administered portion and a participant self-administered portion.

Medication Visits:

At the medication visits, the assessor will update the participant's Contact/Locator form and verify any new numbers. The assessor will complete a Symptom Monitoring Form. If the participant is female, she will be asked if she is currently pregnant. Participants will be asked if they had seen a doctor, visited an emergency room, or if they were hospitalized since their last visit. The assessor will collect information on the participant's current medication use. The assessor will collect a urine sample from the participant, check the urine color, and test all females for pregnancy. As in the longer follow-up assessment visits, sections on Medication Adherence (and count of study pills returned by the participant) and ART Medication History will be administered. The assessor will reinforce the study medication instructions and the medication adherence plan. Participants will be given study medication. At the time of the 6-week medication visit, some participants will also be sent for a fibroscan, if this procedure is indicated based on their FIB-4 score (see section 3.6.D Liver Status).

4.5 QUALITY ASSURANCE

Informed consent quality assurance:

The RA will review Informed Consent Forms (ICFs) for completeness with the participant present. Items to check include, but are not limited to: responses/initials collected for all questions, correct version of ICF used, signed and dated by both participant and RA. The project manager will review ICFs weekly for completeness.

Assessment quality assurance:

Certain quality assurance checks will be built into the assessment. The system will flag any inappropriate responses and prevent the RA from continuing until the issue is resolved.

On the day of the study visit, the research assessor will review the entire completed assessment and address any issues. Within two weeks of completion of the study visit, the data manager or supervisor will review the entire completed assessment and address any issues.

4.6 COMPENSATION

Participants in the trial will receive the equivalent of US $40 in goods or cash for their participation at baseline, 6, 12 and 18 months, which involve the collection of blood for laboratory testing. Participants will also receive compensation equivalent to US $14 (500rub) for short adherence check visits at 6 weeks, 12 weeks, 9 months, and 15 months where medication was dispensed and urine collected for zinc/riboflavin adherence checks. For follow-up assessments that occur on the phone (rather than in person), participants will receive partial (1/2) compensation, as they will not be compensated for travel time or transportation costs, and a phone interview takes less time to complete than an in-person interview. Similar compensation was used in a previous collaborative Russian-Boston research study and was deemed by the PSMU IRB to be an appropriate, non-coercive, amount of funds for involvement in a clinical research project. Participants who refer individuals who enroll in the study will receive 300 rubles (approximately $5USD). Participants who provide updated contact information to research assessors in between research visits (i.e. not during the research assessment) will receive 200 rubles in goods or currency. We will also provide an equivalent of 300 rubles in telephone minutes or other goods or currency for enrollment anniversaries.

4.7 RETENTION

Retention begins at baseline by ensuring that the participant enjoys the experience of participating in the study, by explaining the informed consent and what would happen in the study, and by collecting good contact information. Participants will be asked to provide contact information for 2-3 alternative contacts who may know their whereabouts. Contact numbers will be verified by calling the numbers with the participant present. Participants will be contacted by telephone with appointment reminders and email will be used if one is provided.

The study team will also utilize social networking to connect with participants. If participants can not be reached via phone, in addition to attempting to reach them via text messaging and email, participants will be sent private messages on Vkontakte (Russian social network) utilizing an existing standard script to remind them of their upcoming study visit. No sensitive information will be revealed or ascertained using this method.

Appointment information will be kept up-to-date in the tracking system, so that automatic text messages are accurate. Contact information will be updated at every visit. Study participants will be asked to contact the study team if their phone number changed between study visits; participants will be compensated for this information. All no-shows will be followed up with to reschedule appointments.

Participants will be offered tea, coffee, water, and snacks at each study visit to make their experience in the research study more enjoyable.

In the event that a participant becomes incarcerated during the study period, the participant will not be withdrawn from the study, but kept on a separate list. Incarcerated participants will be called twice per

year in January and August to determine if they have been released and could continue with study procedures.

Additional retention strategies to improve study follow-up:

•The study team will send letters to participants, whom the study team is continually unable to reach, asking them to contact the study team. In the letter, compensation will be offered to participants 300 RUB($5) for calling the study team and updating their contact information. The letter will explicitly state that additional compensation would be received when the participant came in for their scheduled study visit.

•Participants will be paid 200 Russian rubles in goods or currency for providing the study team with updated contact information in between research visits.

•In order to celebrate continued participant engagement, we will provide an equivalent of 300 rub (approximately $5) in telephone minutes or other goods or currency for enrollment anniversaries (i.e. 12-month visit).

•Assessors will wish participants a happy birthday during reminder calls near their date of birth. Participants will also receive sweets when they come in to complete a study visit near the time of major Russian holidays (i.e. New Year, Christmas).

•The team will collaborate with the City Addiction Hospital (CAH) in their retention and follow up efforts. Assessors will be able to contact staff at the CAH to find out if participants were hospitalized there. If participants are found to be hospitalized at the CAH, assessors will be able to receive updated contact information for these participants and contact them directly by phone to schedule an appointment post hospitalization.

Reminders:

One month before each follow-up interview, participants will be contacted by telephone to confirm the date and time of their appointment and to inform them that a call would be made the day before their appointment as a reminder. If a participant cannot be reached (e.g. phone is not in service), an email will be sent to the participant and other participant contacts will be called for updated information.

One week before each follow-up interview, participants will be contacted by telephone to confirm the date and time of their appointment and to inform them that a call would be made the day before their appointment as a reminder. If the participant cannot be reached (e.g. phone is not in service), an email will be sent to the participant and other contacts called for updated information.

72 hours before each follow-up interview, an SMS will be sent to remind the participant of their visit.

24 hours before each follow-up interview if the participant did not respond to SMS, a call will be made to remind the participant of the time and location of their appointment.

If a participant does not show up to the appointment, the participant will be called on the same day to reschedule their appointment. Calls will be made continuously until the participant can be reached.

Contacting participants over the phone is preferred. If participants are not able to be reached by phone, an SMS and email message will be sent to the participant, unless the participant opts out of receiving text messages at baseline.

Note: Confirmation of receipt from participant must be received in order for a message to be considered delivered. If no confirmation is received, it should be assumed that the participant was not reached.

Standard Text for Study Visit Reminders:

SMS: This is a reminder that your visit to Pavlov Medical University is scheduled for____at_____. Please reply to confirm or call 973-53-96 to reschedule.

E-Mail: This is a reminder that your visit to Pavlov Medical University is scheduled for____at_____. Please reply to confirm or call 973-53-96 to reschedule.

5. ASSESSMENTS

5.1 BASELINE ASSESSMENT

The baseline assessment will be conducted immediately following the screening, informed consent, and blood draw. Assessment will be interviewer-administered with the exception of sections deemed to ask sensitive questions, which will be self-administered by the participant. At baseline the following surveys will be administered: Demographics[1]; HIV Testing and HCV Diagnosis[2]; Co-Morbidities[3]; Russian Brief Pain Inventory[4]; Medications; Zinc intake[5], HIV Symptom Index[6]; The Center for Epidemiologic Studies Depression Scale (CES-D) [7,8]; Emotional Health: International Personality Item Pool (IPIP) and the Revised Life Orientation Test (LOT-R)[9,10]; The Fagerström Test for Nicotine Dependence[11,12]; Alcohol Surrogates[13]; MINI: Alcohol Dependence/Abuse[14]; 30 Day Timeline Follow Back[15]; Drug Use (modified Risk Behavior Survey)[16,17]; 24 Hour Activities; Social Support Scale[18]; and VR-12 Health Survey.[19,20]

5.2 FOLLOW UP ASSESSMENTS

The 6-, 12- and 18-month ZINC assessments contain the same sections as the baseline assessment with the addition of questions on Medication Adherence[21,22]; Medication Satisfaction[23]; ART Use and Adherence[21]; and Opportunistic Infections[24]. The MINI: Alcohol Dependence/Abuse section are omitted from 6-, 12- and 18-month assessments. Questions on Medication Adherence[21] and Medication Satisfaction[23] are also asked at shorter medication visits.

5.2.A. MEDICATION VISITS ASSESSMENTS

Medication symptoms and questions on Medication Adherence[21] and Medication Satisfaction[23] are also asked at shorter medication visits.

6. PARTICIPANT SAFETY

ZINC AE/SAE Flow Chart

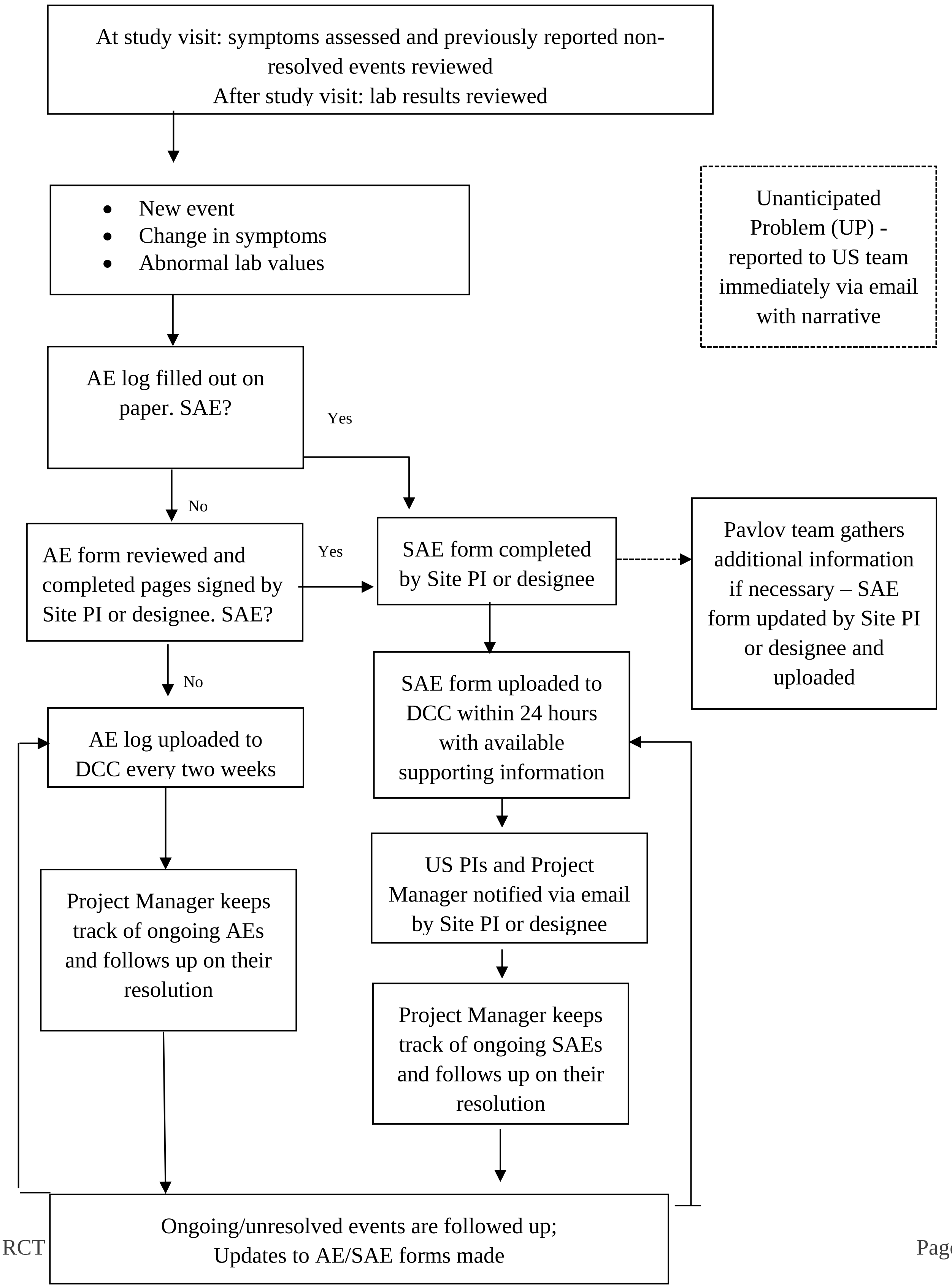

6.1 SPECIFICATION OF SAFETY PARAMETERS

An **Adverse Event (AE)** is defined as any untoward or unfavorable medical occurrence in a human subject, including any abnormal sign (for example, abnormal physical exam or laboratory finding), symptom, or disease, temporally associated with the subject's participation in the research, whether or not considered related to the subject's participation in the research.

An AE can therefore be any new sign, reaction, symptom, event, disease or a worsening in frequency or severity of a preexisting condition that occurs during the course of the study.

Stable chronic conditions that were present prior to study entry and do not worsen are not considered AEs.

SERIOUS Adverse Event – for an event to be defined as serious it will be Grade 1-6 below. Grade 0 would be "not serious".

Grade (1) results in death;

Grade (2) is life-threatening (places the subject at immediate risk of death from the event as it occurred);

Grade (3) results in inpatient hospitalization or prolongation of existing hospitalization;

Grade (4) results in a persistent or significant disability/incapacity;

Grade (5) results in a congenital anomaly/birth defect; or

Grade (6) based upon appropriate medical judgment, may jeopardize the subject's health and may require medical or surgical intervention to prevent one of the other outcomes listed in this definition (examples of such events include allergic bronchospasm requiring intensive treatment in the emergency room or at home, blood dyscrasias or convulsions that do not result in inpatient hospitalization, or the development of drug dependency or drug abuse).

Unanticipated Problem- for an event to be an Unanticipated Problem it must

be unexpected AND

be related or possibly related to participation in the research AND

suggest that the research places subjects or others at a greater risk of harm (including physical, psychological, economic, or social harm) than was previously known or recognized. OR meet the definition of SERIOUS

Suspected Adverse Drug Reaction – Any adverse event for which there is a reasonable possibility that the drug caused the adverse event. Reasonable possibility means there is evidence to suspect a causal relationship. It is considered unexpected if it is not consistent with the risk information described in the

general investigational plan. A suspected adverse drug reaction will be defined as a recorded adverse event that is unexpected and deemed to be possibly, probably, or definitely related to the study drug.

6.2 THE METHODS AND TIME FOR ASSESSING, RECORDING, AND ANALYZING SAFETY PARAMETERS

- Participant symptoms will be assessed at baseline to document any chronic conditions or symptoms that existed prior to introduction of study medication. These will be documented on the Baseline AE log. This list will be reviewed and compared to reported events throughout the study. *If the participant reports the same ongoing symptom (same severity) during subsequent visits, the symptom is not recorded as an Adverse Event (AE). If the event is new (not previously reported) or worsened, as determined by assessor, then the AE should be reported.*

- During each scheduled visit, the assessor will ask the participant how they feel and review the list of symptoms of concern (starting with the symptoms recorded at the previous visit). Any event that meets the above criteria for an AE/SAE/UP should be recorded. In the case of unresolved AEs, clinical staff will update the AE log with any follow-up information that will be gathered during their investigation.

- The site will receive the results of all blood work performed on study participants from the designated lab. If lab results meet the criteria described in the protocol as an AE and are considered clinically significant by the site clinician then an AE should be recorded. **Please see Box 1.**

    - Participants will be alerted of abnormal lab results and receive a recommendation to see their local provider. All abnormal lab results obtained at the baseline visit will be listed on the Baseline AE log. During follow-up visits, abnormal lab results will be listed as an AE *only if* the abnormal lab results:
        - Developed at follow-up (i.e. were not previously recorded at baseline).
        - Worsened in severity than what was previously recorded at baseline.
        - Or considered to be clinically significant.

- All AEs will be assessed to determine if they meet criteria for a Serious Adverse Event (SAE). If the AE is serious, then the SAE form is completed and appropriate reporting measures followed (see below). Investigators are encouraged to consult with the US Team, if they are uncertain how to classify an event.
- The list of participant's current medications will be reviewed and updated at every study visit, starting at baseline.
- If an event is discovered outside of the scheduled study visits, it should still be recorded accordingly.
- Action Taken will be determined by the assessors for all AEs that are Mild and Moderate and by Site PI or designee for SAEs and AEs that are severe, life-threatening or fatal.

Box 1. Abnormal Lab Results

	A. Normal Lab Values
**Cholesterol (total)**	to 5.7 mmol/L<br>*[220 mg/dL]*
**HDL**	1.04 – 1.55 mmol/L<br>*[40 – 60 mg/dL]*
**HS CRP**	to 10 mg/L
**CD4**	35.0-55.0% (from lymphocytes)
**HIV-1 RNA**	> 1,000 copies
**Hemoglobin**	M: 130-160 g/L *[13-16 g/dL]*<br>F: 120-140 g/L *[12-14 g/dL]*
**Platelet**	180 – 320 x 10^9/L<br>*[180,000-320,000/uL]*
**eGFR (Crea)**	M: 53-97 mmol/L *[0.6-1.1mg/dL]*<br>F: 44-80 mmol/L *[0.5-0.9mg/dL]*
**HCV Ag qualitative**	negative
**HCV Ab**	negative
**AST**	M: to 37 units/L<br>F: to 31 units/L
**ALT**	M: to 40 units/L<br>F: to 31 units/L

1. Laboratory results reviewed by study clinician and abnormal results (outside of normal values listed in column A) are identified.

2. If clinician determines abnormal lab results to be clinically significant, participant contacted by phone: result shared and participant referred to local provider.

3. Abnormal result registered as an AE.

4. If clinician determines abnormal lab results to be not clinically significant (for example slightly elevated AST and ALT in case of chronic hep C), result shared with participant during their next study visit, but not recorded as an AE.

5. In situations where laboratory values were deemed dangerously abnormal by study clinician, participant is alerted and asked to call emergency services.

6. As per Pavlov protocol, all participants will be provided with pre- and post-test HCV counseling. Post-test counseling will take place at the time of their next study visit.

6.3 PROCEDURES FOR ELICITING REPORTS OF AND FOR RECORDING AND REPORTING ADVERSE EVENT AND INTERCURRENT ILLNESSES

The following information should be present to complete AE and SAE forms during the initial report (on the day of finding out about the event):

- Description of the event
- Date of onset and resolution (if known)
- Severity
- Assessment of expectedness (is the event anticipated in terms of nature, severity, or frequency) given (a) the research procedures that are described in the IRB protocol and informed consent document; and (b) the characteristics of the subject population being studied
- Assessment of relatedness to study drug
- Any actions taken

Following the initial report, additional information may need to be gathered to complete the AE and SAE forms and to evaluate the event for relatedness. This process may include obtaining hospital discharge reports, physician records, autopsy records or any other type of records or information necessary to provide a complete and clear picture of the SAE and events preceding and following the event.

SAE Reporting:

If the SAE is not resolved or stabilized at this time or new information becomes available after the SAE form is completed the SAE form should be updated as soon as possible. Any changes or updates to the SAE form must be re-reviewed and re-authorized by the study clinician.

The site must actively seek information about the SAE until the SAE is resolved, stabilized or until the participant is lost to follow-up and terminated from the study.

<u>To summarize</u>: upon determining an Adverse Event is Serious, the following procedures must be followed:

- The study staff, while meeting/talking with the participant or person providing details on the event, gather as much information about the event from the participant as possible and complete the appropriate forms.

- The completed AE and SAE forms are reviewed by key personnel on the Pavlov team (i.e. Site PI or designee). Any relevant clinical documents (labs, physician notes) available at that time are provided to key personnel on the Pavlov team (i.e. Site PI or designee) within 24 hours of finding out about the event.

- After initial notification, the SAE is updated with any additional information.

All unanticipated problems (UPs) must be reported to the US team immediately. The US team must report all UPs to the BUMC IRB and NIAAA within 48 hours of discovering their occurrence.

AEs and SAEs will be reported to the URBAN ARCH DSMB every six months.

6.4 THE TYPE AND DURATION OF THE FOLLOW-UP OF PARTICIPANTS AFTER ADVERSE EVENTS

All non-mild adverse events (including serious adverse events) will be followed until the event is resolved, stabilized, or until the end of individual's participation in the study.

Site PI or designee will determine a follow-up plan on a case-by-case basis based on their clinical judgment.

6.5 UNBLINDING PROTOCOL

Participants could be unblinded in the event of an urgent medical need, as determined by the clinician evaluating the participant.

The following are examples of events that may result in emergency unblinding:

-An SAE occurs that is thought to be most likely or definitely related to the study drug.

-An AE or SAE occurs and the clinician treating the patient concludes that knowledge of the treatment arm is necessary to determine the therapy provided to the patient.

-The study drug is accidentally ingested by a child.

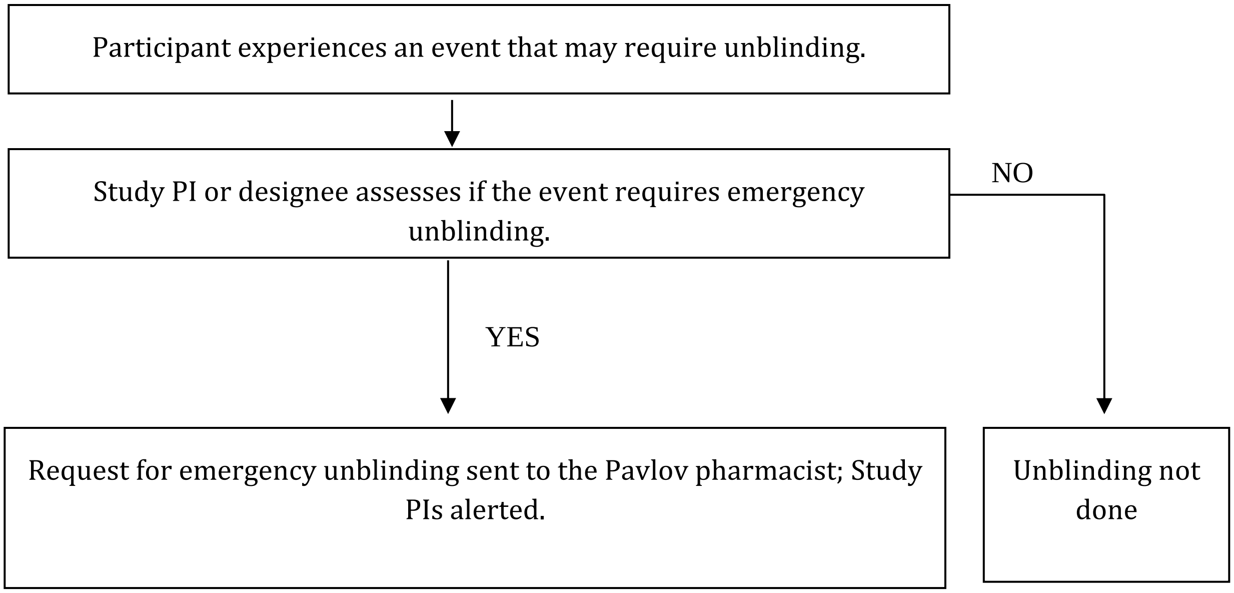

6.6 DATA SAFETY AND MONITORING BOARD

The Data and Safety Monitoring Board (DSMB) was set up to monitor the URBAN ARCH Cohort studies (i.e., Uganda, Russia, Boston) and Intervention trials (i.e., ZINC). The DSMB serves in an advisory capacity to the study PIs and the National Institute on Alcohol Abuse and Alcoholism (NIAAA) to monitor participant safety, data quality and evaluate the progress of the studies conducted under the URBAN ARCH consortium funded by NIAAA.

The DSMB is responsible for ensuring participant safety (by reviewing blinded and unblinded safety data on a regular basis and assessing the safety of study procedures) and for monitoring the overall conduct of the study.

The DSMB will be required to provide recommendations about starting, continuing, temporarily or permanently suspending the trial. In addition, the DSMB will be asked to make recommendations, as appropriate, about:

- Benefit/risk ratio of procedures and participant burden
- Selection, recruitment, and retention of participants
- Protocol violations and adherence to protocol requirements
- Completeness, quality, and analysis of measurements
- Amendments to the study protocol and consent forms
- Participant safety
- Notification of and referral for abnormal findings

7. DATA MANAGEMENT

7.1 DATA COLLECTION

The majority of study data will be captured electronically via a secure, web-based data capture system with the exception of: TLFB data, which will be collected on paper calendars and data on drug and alcohol use disorders, which will be captured on paper, scanned and read in using TELEform software.

7.2 QUALITY CONTROL PROCESS

Quality control measures will include: detailed and unambiguous specifications for completion of data forms, including rules for coding skipped questions and missing data, training of study staff responsible for data collection and built-in validation rules, error checks, question skips for electronic data capture, and computer algorithms to check for out-of-range codes and internal inconsistencies. All data, regardless of capture method, will be converted to SAS datasets and reviewed for logic, skip patterns, response ranges, out-of-range codes, and internal inconsistencies. The RAs will be queried monthly regarding any noted inconsistencies.

7.3 DATA SECURITY AND CONFIDENTIALITY

Screening forms and most other research paperwork will not include the participant's name. Instead a unique ID will be assigned to each person screened; then another number will be assigned to those who enrolled. There will be a master list of names and identification numbers. The master list of study participants will be destroyed seven years after the completion of study analyses and publication of all study manuscripts. Any documents with identifiable participant data will be accessible only to the Russian Co-Investigators, the Russian project manager, and the RA assessors who will be recruiting and following participants.

Tracking information will be kept similarly. Computer data will be password protected, and accessible only to those needing the information for follow-up purposes.

The BDM Core of the URBAN ARCH Consortium designed, developed and will maintain the electronic data collection forms, participant and data tracking, and underlying SQL database systems, and will implement procedures for data quality control, including multiple checks for entered data. Electronic data collection forms are designed to read easily, have clear instructions, preprogrammed skip patterns, real-time range checks and internal logic to minimize missing data and resulting in "cleaner" data at capture. The website and accompanying database are located on secure, password-protected servers, behind the BU firewalls. The BDM Core has access to two Unix servers, including a Linux Beowulf cluster currently configured

FORM	STUDY VISIT

with 118 CPUs, as well as an SMP Linux server with 4 x Six-Core AMD Opteron processors (a total of 24 cores x 2.4 GHz each), 64 GB of RAM, and 6 TB (4TB usable) storage capacity. Additionally, the DCC has three dedicated servers, all of which are dual processors with 150 gigabytes for data storage: an SQL database server; a server used for Web site development and management, running Internet Information Server for web page hosting; and a server used for web development pre-production testing environment. The web and database servers use Secure Socket Layering (SSL) to ensure data security and confidentiality. Two fax servers and additional server and a flatbed scanner comprise the Teleform® system. Servers incorporated RAID hard drives for data redundancy. A separate web server dedicated for Cold Fusion applications is also available.

7.4 WEB SYSTEMS

The study will use two web systems: a computerized tracking system and CASIC (for assessment). The computerized tracking system will contain all participant tracking details. This system is web-based, allowing multiple users to access the system. Study forms will be completed according to the schedule below.

	Screen A	Screen B & Baseline Visit		6-Week	12-Week	6-Month	9-Month	12-Month	15-Month	18-Month	As Needed
		Screen B	Baseline								
Screener A	X										
Screener B		X									
Consent and enrollment form			X								
Contact info			X	X	X	X	X	X	X	X	
Phlebotomy form			X			X		X		X	
Processing form Pasteur			X			X		X		X	
Processing form IBS			X							X	
Assessment (CASIC and Web)			X	X	X	X	X	X	X	X	
Randomization Page			X								
Medication visit checklist (CASIC)			X	X	X	X	X	X	X	X	
Baseline Symptom Monitoring (CASIC)			X								
Medication collection (Paper)			X	X	X	X	X	X	X	X	
Baseline tracking form			X								
Symptom monitoring (Follow-up) (CASIC)				X	X	X	X	X	X	X	
Follow-up tracking form				X	X	X	X	X	X	X	
Participant tracking overview											X
Study conclusion form											X
AE/SAE form											X
Pre-enrollment contact log											X
Contact log											X

Electronic forms to be completed at screening and enrollment:

- Screening Consent: The script that the research assessor reads to potential participants, describing the purpose of the study and what would occur during the screening.
- Screener A: This will be completed once a participant gives verbal consent to be screened. It confirms participant eligibility for the study.
- Screener B: This will be completed at the baseline visit to confirm that the participant is HIV-positive, ART-naïve and not pregnant or breastfeeding. This requires the RA to verify the participant's HIV status (either past HERMITAGE participant; HIV/VL/CD4 test result; medical records/summary page; doctor's letter). The participant will be asked if they are currently taking or had ever taken ART. A participant's ART naïve status will require documentation from the participant's medical record/summary page or a doctor's letter.

Electronic forms/documents just for those who are enrolled:

- Informed Consent: A copy will be maintained at PSMU and the participant will receive a copy.

- Consent and Enrollment Tracking Form: This will be completed in the presence of the participant, after the informed consent is signed. It will generate the participant ID number. This form will allow the assessor to confirm that the participant was enrolled and to enter the answers to the permissions marked by the participant on the consent form (storage of blood for clinical, virological, and immunological studies related to HIV disease and other associated illnesses; contact to use blood for studies not listed above; post-study contact for future research).
- Contact Information/Locator Form: This form will contain the phone number, email address, and physical address of the participant and several additional friends or family contacts to facilitate the follow-up process (the minimum required information is the contact details for the participant and 2 friends or family members). If the participant cannot give the required contact information they must be unenrolled from the study. Assessor will confirm that the phone numbers provided are valid in the presence of the participant.

Tracking system

Forms

- Baseline Tracking Form: This will be completed on the day of the baseline visit, after the baseline assessment is administered to the participant. It will track that the participant was sent for a blood draw and that a resource card and compensation was provided to the participant.
- Follow up Tracking Forms: This will be completed on the day of each follow up assessment after the assessment is administered to the participant. It will track the completion of the follow up assessment, blood draw, and compensation.
- Participant Tracking Overview: This will provide an overview of all assessments for a participant on one page. The only field that must be filled in by the assessor is the scheduled date of assessment (if that date changes during one of the reminder calls), all other information will be pulled from previously completed forms.

8. STATISTICAL ANALYSIS

8.1 PRIMARY ANALYSES

The study aims to test the hypothesis that compared with placebo, participants receiving zinc supplementation will have significantly 1) lower VACS index scores; 2) higher CD4 cell counts; 3) lower Reynolds risk score and; 4) lower biomarker levels of microbial translocation and inflammation. The primary outcome is improved markers of mortality, as measured by change in VACS index score between baseline and 18 months. The secondary outcomes are slower HIV disease progression, as measured by change in CD4 cell count; improved markers of AMI risk, as measured by the Reynolds Risk Score; and lower biomarker levels of microbial translocation and inflammation.

This study will be conducted under the intention-to-treat principle and thus main analyses will include all participants according to their randomized assignment. Descriptive statistics will be calculated for variables at baseline and each follow-up time point. At baseline, participant characteristics will be

presented by randomized arm to assess whether there are any differences between groups. Spearman correlation coefficients will be obtained to identify pairs of variables that may be collinear (r>0.4) and would therefore not be included together in regression analyses.

The main analysis evaluating the impact of zinc on the primary study outcome (i.e., change in VACS index) will use multiple regression models that include randomization group (i.e., zinc vs. placebo) as the main independent variable. The regression analyses will control for the two block randomization stratification factors: heavy alcohol consumption during the past week and gender. In addition, the models will control for baseline characteristics that differ between groups in order to avoid confounding. Potential confounders of interest, measured at baseline, include demographics, past month alcohol use, age, gender, anti-inflammatory medication use, cardiovascular disease risk factors, HCV status, substance use (e.g., alcohol, smoking, cocaine), CD4 cell count, HIV-1 RNA, duration of awareness of HIV infection, and socioeconomic status. If the data are normally distributed, multiple linear regression models will be used. However, if the distribution is skewed, transformations of the data will be performed (e.g., log transformation). If an appropriate transformation is not identified, a median regression model will be used. The secondary outcomes, including HIV disease progression, as measured by change in CD4 cell count (Aim 2); the Reynolds risk score (Aim 3); and biomarkers of microbial translocation and inflammation (Aim 4) will be analyzed using the same approach described above. A secondary analysis will be conducted using a per protocol approach that includes only those participants who were adherent with their assigned intervention (i.e., taking zinc or placebo 80% of the time).

8.2 ADDITIONAL EXPLORATORY ANALYSES

Effect of zinc over time: The main analyses will focus on the VACS index, our primary outcome, and other secondary outcomes at the 18 month time point as the primary interest is in evaluating the long-term effects of zinc and it is anticipated that effects will change over time. Additional analyses using generalized linear mixed effects models will be used to incorporate the repeated measures for each outcome in the same model and will test for possible zinc by time interactions (e.g. does the effect of zinc increase over time). The mixed effects models will include subject-specific random intercepts and slopes to account for the correlation due to having repeated observations for each subject. The mixed models will be implemented in SAS PROC MIXED. In addition to the mixed effects model, we will also consider additional confirmatory models such as the general linear model for correlated data using an autoregressive covariance structure.

Moderators and Mediators: Additional analyses will also be conducted to evaluate whether the effects of zinc depend on level of alcohol consumption. Models will be fit including a zinc by heavy alcohol interaction term. If the interaction is significant, subsequent stratified analyses will be conducted to evaluate the effect of zinc in each alcohol group. Given the potentially complex role of ART use in the relationship between zinc and each outcome, various analyses will be considered to explore its possible role. First, it is not expected that initiation of ART use during follow-up to differ by randomized arm, however exploratory analyses will be conducted to compare initiation of ART use by randomized group. In addition, longitudinal analyses will be conducted including ART use as a time dependent variable in

order to explore a potential zinc by ART use interaction. If ART use does not appear to be an effect modifier, we will also include it as time will also be included as a dependent covariate to evaluate whether it is a confounder. Lastly, to evaluate ART use as a potential mediator of the relationship between zinc and outcomes of interest, the approach described by Baron and Kenny will be used. To evaluate mediation, the change in magnitude of the zinc effect on our primary outcome, the VACS index (and our other secondary outcomes) in models with and without adjustment for ART use will be focused on. Results will be confirmed using structural equation models (SEMs) to simultaneously model the hypothesized mediated relationships. Fit statistics such as the root mean square error of approximation (RMSEA), Comparative Fit Index (CFI), and the chi-square statistic, will be presented to evaluate the goodness of fit of the SEMs.

Enrollment and Attrition/Missing data: Subjects who meet eligibility criteria and agree to participate will be compared with subjects who were determined to be eligible but declined enrollment on data captured during eligibility assessment. The 2 independent samples t-test and Fisher’s exact test will be used to test for statistically significant differences between subjects who enroll and those who do not, and to test for significant differences between subjects lost to follow-up and those who complete it. Missing data patterns will be evaluated including the frequency and percentage of subjects missing for each variable and the distribution of the number of variables missing for subjects. In addition, data collected to the point of lost to follow-up will be compared to the data of those who complete the study to examine missing data mechanisms, e.g., missing completely at random (MCAR), missing at random (MAR), or not missing at random (NMAR). We will consider various approaches to account for missing data such as multiple imputation methods and likelihood-based approaches, if needed. In situations where missing data occurs, we will document the reasons for the missing data whenever possible. The proposed study has accounted for a 20% random noninformative loss to follow-up and will still have sufficient power with this potential loss in size.

9. STAFF TRAINING

All study staff will be trained on the study protocol, including administration of study medication, symptom monitoring, and participant assessment prior to initiation of recruitment and enrollment. Training took place in-person in St. Petersburg and via webinars.

10. STUDY CONTACTS

This study was led by two US PIs: Dr. Samet and Dr. Freiberg.

REFERENCES

1. McLellan AT, Luborsky L, Cacciola J, Griffith J, Evans F, Barr HL, O'Brien CP. New data from the Addiction Severity Index. Reliability and validity in three centers. *J Nerv Ment Dis*. 1985;173(7):412-423.

2. National Institute on Drug Abuse: Seek, Test, Treat and Retain Initiative. HIV/HCV/STI Testing Status and Organizational Testing Practices Questionnaire. 2011.

3. Kazis LE, Miller DR, Clark J, Skinner K, Lee A, Rogers W, Spiro A,3rd, Payne S, Fincke G, Selim A, Linzer M. Health-related quality of life in patients served by the Department of Veterans Affairs: results from the Veterans Health Study. *Arch Intern Med*. 1998;158(6):626-632.

4. Kalyadina SA, Ionova TI, Ivanova MO, Uspenskaya OS, Kishtovich AV, Mendoza TR, Guo H, Novik A, Cleeland CS, Wang XS. Russian Brief Pain Inventory: validation and application in cancer pain. *J Pain Symptom Manage*. 2008;35(1):95-102.

5. Lacey JM. Zinc-specific food frequency questionnaire. *Can J Diet Pract Res*. 2007;68(3):150-152.

6. Justice AC, Holmes W, Gifford AL, Rabeneck L, Zackin R, Sinclair G, Weissman S, Neidig J, Marcus C, Chesney M, Cohn SE, Wu AW. Development and validation of a self-completed HIV symptom index. *J Clin Epidemiol*. 2001;54 Suppl 1:S77-90.

7. Radloff LS. The CES-D scale: a self-report depression scale for research in the general population. *Applied Psychological Measurement*. 1977;1:385-401.

8. Chishinga N, Kinyanda E, Weiss HA, Patel V, Ayles H, Seedat S. Validation of brief screening tools for depressive and alcohol use disorders among TB and HIV patients in primary care in Zambia. *BMC Psychiatry*. 2011;11:75. PMCID: PMC3112078.

9. Goldberg L, Johnson J, Eber H, Hogan R, Ashton M, Cloninger C, Gough H. The international personality item pool and the future of public-domain personality measures. *Journal of Research in Personality*. 2006;40:84.

10. Scheier MF, Carver CS, Bridges MW. Distinguishing optimism from neuroticism (and trait anxiety, self-mastery, and self-esteem): a reevaluation of the Life Orientation Test. *J Pers Soc Psychol*. 1994;67(6):1063-1078.

11. Heatherton TF, Kozlowski LT, Frecker RC, Fagerstrom KO. The Fagerstrom Test for Nicotine Dependence: a revision of the Fagerstrom Tolerance Questionnaire. *Br J Addict*. 1991;86(9):1119-1127.

12. Meneses-Gaya IC, Zuardi AW, Loureiro SR, Crippa JA. Psychometric properties of the Fagerstrom Test for Nicotine Dependence. *J Bras Pneumol*. 2009;35(1):73-82.

13. Leon DA, Saburova L, Tomkins S, Andreev E, Kiryanov N, McKee M, Shkolnikov VM. Hazardous alcohol drinking and premature mortality in Russia: a population based case-control study. *Lancet*. 2007;369(9578):2001-2009.

14. Sheehan DV, Lecrubier Y, Sheehan KH, Amorim P, Janavs J, Weiller E, Hergueta T, Baker R, Dunbar GC. The Mini-International Neuropsychiatric Interview (M.I.N.I.): the development and validation of a structured diagnostic psychiatric interview for DSM-IV and ICD-10. *J Clin Psychiatry*. 1998;59 Suppl 20:22-33;quiz 34-57.

15. Sobell LC, Sobell MB. *Alcohol Timeline Followback (TLFB) Users' Manual.* Toronto, Canada: Addiction Research Foundation; 1995.

16. Weatherby N, Needle R, Cesar H, Booth R, McCoy C, Watters J, Williams M, Chitwood D. Validity of self-reported drug use among injection drug users and crack smokers recruited through street outreach. *Eval Program Plann*. 1994;17:347-347-355.

17. Needle R, Fisher DG, Weatherby N, Chitwood D, Brown B, Cesari H, Booth R, Williams ML, Watters J, Andersen M, Braunstein M. The reliability of self-reported HIV risk behaviors of drug users. *Psychol Addict Behav*. 1995;9:242-250.

18. Fleishman JA, Sherbourne CD, Crystal S, Collins RL, Marshall GN, Kelly M, Bozzette SA, Shapiro MF, Hays RD. Coping, conflictual social interactions, social support, and mood among HIV-infected persons. HCSUS Consortium. *Am J Community Psychol*. 2000;28(4):421-453.

19. Kazis LE, Miller DR, Clark JA, Skinner KM, Lee A, Ren XS, Spiro A,3rd, Rogers WH, Ware JE,Jr. Improving the response choices on the veterans SF-36 health survey role functioning scales: results from the Veterans Health Study. *J Ambul Care Manage*. 2004;27(3):263-280.

20. Wu AW, Revicki DA, Jacobson D, Malitz FE. Evidence for reliability, validity and usefulness of the Medical Outcomes Study HIV Health Survey (MOS-HIV). *Qual Life Res*. 1997;6(6):481-493.

21. Chesney MA, Ickovics JR, Chambers DB, Gifford AL, Neidig J, Zwickl B, Wu AW. Self-reported adherence to antiretroviral medications among participants in HIV clinical trials: the AACTG adherence instruments. Patient Care Committee & Adherence Working Group of the Outcomes Committee of the Adult AIDS Clinical Trials Group (AACTG). *AIDS Care*. 2000;12(3):255-266.

22. Walsh JC, Mandalia S, Gazzard BG. Responses to a 1 month self-report on adherence to antiretroviral therapy are consistent with electronic data and virological treatment outcome. *AIDS*. 2002;16(2):269-277.

23. Atkinson MJ, Sinha A, Hass SL, Colman SS, Kumar RN, Brod M, Rowland CR. Validation of a general measure of treatment satisfaction, the Treatment Satisfaction Questionnaire for Medication (TSQM), using a national panel study of chronic disease. *Health Qual Life Outcomes*. 2004;2:12. PMCID: PMC398419.

24. Cunningham WE, Sohler NL, Tobias C, Drainoni M, Bradford J, Davis C, Cabral HJ, Cunningham CO, Eldred LDH, Wong MD. Health Services Utilization for People with HIV Infection: Comparison of a Population Targeted for Outreach with the U.S. Population in Care. *Med Care*. 2006;44(11):1038-1047.

STATISTICAL ANALYSIS PLAN

Zinc for INflammation and Chronic disease in HIV (ZINC HIV)

Principal Investigators:

Jeffrey H. Samet, MD, MA, MPH – Boston Medical Center (contact)

Matthew S. Freiberg, MD, MSc - Vanderbilt University Medical Center

Study Statistician: Debbie M. Cheng, ScD – Boston University School of Public Health

NIAAA Award Number: U01AA021989

Clinicaltrials.gov Registration: NCT01934803

Boston University Medical Campus IRB Protocol Number: H-31901

Enrollment Dates: October 2013 – June 2015

Statistical Analysis Plan (SAP) Version Date: 10/26/2018

SAP Revision History

**Date of revision**	**Section number changed**	**Description and reason for change**
6/8/2017	5.3 Missing Data	Updating multiple imputation plan to clarify details on specific variables will be included in imputation model.

8/15/2017	5.3 Missing Data	Adding predictors for missing outcome data.
1/26/2018	5.1 Outcome Definitions	Posthoc replacement of LPS with IFABP another marker of microbial translocation, due to inability to conduct LPS testing.
2/28/2018	5.5 Harms	Adding posthoc plans for mortality analyses using actual mortality events.
4/13/2018	5.4 Additional Analyses	Adding posthoc plans for analyzing individual VACS components.
10/26/2018	5.1 Outcome Definitions	Replacing 16srDNA with LBP, due to the lab having trouble extracting rDNA from study samples.

SECTION 1: INTRODUCTION

1.1 STUDY HYPOTHESES

We hypothesize that as compared with placebo, participants receiving zinc supplementation will have significantly:

Hypothesis 1- Smaller change in VACS (Primary);

Hypothesis 2- Greater change in CD4 cell counts (Secondary);

Hypothesis 3- Lower Reynolds risk score (Secondary);

Hypothesis 4- Lower biomarker levels of microbial translocation and inflammation (Secondary).

SECTION 2: STUDY METHODS

2.1 STUDY DESIGN

ZINC is a double-blinded randomized placebo-controlled trial of zinc supplementation (Zinc for INflammation and Chronic disease in HIV [ZINC]) among HIV-positive heavy drinkers in Russia to evaluate the efficacy of zinc to 1) improve markers of mortality, as measured by the VACS index; 2) slow HIV disease progression, as measured by CD4 cell count; 3) improve markers of coronary heart disease (CHD) risk, as measured by the Reynolds risk score and; 4) decrease microbial translocation and inflammation, as measured by serum biomarkers. Participants will receive study medication over 18 months, with study visits occurring at 6, 12, and 18 months post enrollment, and shorter medication adherence visits at 6 weeks, 12 weeks, 9 months, and 15 months. ZINC RCT is nested within the Russia ARCH cohort of the Uganda, Russia, Boston Alcohol Network for Alcohol Research Collaboration on HIV/AIDS (URBAN ARCH) Consortium, which aims to understand how alcohol use impacts people affected by HIV and develop interventions to reduce alcohol use and alcohol and HIV-related consequences in this population.

2.2 RANDOMIZATION

See section 3.2 of Study Protocol

2.3 SAMPLE SIZE

See section 2.7.A. of Study Protocol

2.4 FRAMEWORK

This study will use a superiority hypothesis testing framework. We will test whether zinc: 1) improves markers of mortality, as measured by the VACS index; 2) slows HIV disease progression, as measured by CD4 cell count; 3) improves markers of coronary heart disease (CHD) risk, as measured by the Reynolds risk score and; 4) decreases microbial translocation and inflammation, as measured by serum biomarkers, compared to placebo.

2.5 STATISTICAL INTERIM ANALYSES AND STOPPING GUIDANCE

None

2.6 TIMING OF FINAL ANALYSES

All outcomes analyzed collectively once all 18-month outcome data have been collected and cleaned.

2.7 TIMING OF OUTCOME ASSESSMENTS

See section 3.5A of Study Protocol

SECTION 3: STATISTICAL PRINCIPLES

3.1 Confidence intervals and P values

Statistical tests will be 2-sided and will be performed using a 5% significance test. Confidence intervals will be reported for measures of effect.

3.2 Adherence and protocol deviations

Participants will be considered adherent to study intervention if they self-report $\geq$80% adherence on the visual analog scale (VAS) for $\geq$ three study visits. Descriptive statistics on the percent of participants adherent will be summarized by randomized group.

3.3 Analysis Populations

The intention-to-treat (ITT) population will include all randomized participants according to the study group assigned.

The per-protocol population will include all participants meeting the definition of adherence noted in section 3.2 above.

SECTION 4: TRIAL POPULATION

4.1 Screening Data

The following data will be provided for all screened participants: number of patients assessed for eligibility, number of participants enrolled, reasons for ineligibility and non-enrollment.

4.2 Eligibility

See section 2.5 of Study Protocol.

4.3 Recruitment

The CONSORT diagram will present data on number of participants screened, eligible, enrolled, randomized, assigned to each study arm, and completing follow up.

4.4 Withdrawal/follow up

Reasons for discontinued treatment will be presented in the CONSORT diagram/trial profile.

4.5 Baseline Participant Characteristics

Descriptive statistics will be calculated for the following variables at baseline overall and stratified by randomized group: age, sex, heavy alcohol (past week), education, marital status, employment, VACS Index, CD4, Reynolds risk score, IL-6, D-dimer, sCD14, 16sRDNA , smoking status (current, ever, never), BMI, CVD, AST/ALT, FIB4, HCV (test), HVL, hemoglobin, platelets, eGFR, Blood pressure, Total cholesterol, HDL cholesterol, CRP, mather/father MI. For continuous variables, the following will be provided: median, mean, standard deviation, 0th, 25th, 50th, 75th, and 100th percentiles. For categorical variables, frequencies and proportions will be provided. For each primary and secondary outcome, descriptives will also be reported stratified by arm and follow-up time, (note, no testing will be done for any of the above).

SECTION 5: ANALYSIS

5.1 Outcome Definitions

**Primary (Aim 1)**	VACS Index (change from baseline to 18 months)				
**Secondary (Aim 2)**	CD4 (change from baseline to 18 months)				
**Secondary (Aim 3)**	Reynolds Risk Score 18 months)				
**Secondary (Aim 4)**	IL-6 (18 months)	D-dimer (18 months)	sCD14 (18 months)	IFABP (18 months)	LBP (18 months)
**Secondary:** repeated measures of all available follow-up data for each outcome					
**Secondary:** change from baseline to 18 months					

See section 2.1 of Study Protocol.

5.2 Analysis Methods

The study aims to test the hypothesis that compared with placebo, participants receiving zinc supplementation will have significantly 1) lower VACS index scores; 2) higher CD4 cell counts; 3) lower Reynolds risk score and; 4) lower biomarker levels of microbial translocation and inflammation. The primary outcome is improved markers of mortality, as measured by change in VACS index score between baseline and 18 months. The secondary outcomes are slower HIV

disease progression, as measured by change in CD4 cell count; improved markers of AMI risk, as measured by the Reynolds Risk Score; and lower biomarker levels of microbial translocation and inflammation.

This study will be conducted under the intention-to-treat principle and thus main analyses will include all participants according to their randomized assignment. Descriptive statistics will be calculated for variables at baseline and each follow-up time point. At baseline, participant characteristics will be presented by randomized arm to assess whether there are any differences between groups. Spearman correlation coefficients will be obtained to identify pairs of variables that may be collinear (r>0.4) and would therefore not be included together in regression analyses.

The main analysis evaluating the impact of zinc on the primary study outcome (i.e., change in VACS index) will use multiple regression models that include randomization group (i.e., zinc vs. placebo) as the main independent variable. The regression analyses will control for the two block randomization stratification factors: heavy alcohol consumption during the past week and gender. In addition, the models will control for baseline characteristics that differ between groups in order to avoid confounding. Potential confounders of interest, measured at baseline, include demographics, past month alcohol use, age, gender, anti-inflammatory medication use, cardiovascular disease risk factors, HCV status, substance use (e.g., alcohol, smoking, cocaine), CD4 cell count, HIV-1 RNA, duration of awareness of HIV infection, and socioeconomic status. If the data are normally distributed, multiple linear regression models will be used. However, if the distribution is skewed, transformations of the data will be performed (e.g., log transformation). If an appropriate transformation is not identified, a median regression model will be used. The secondary outcomes, including HIV disease progression, as measured by change in CD4 cell count (Aim 2); the Reynolds risk score (Aim 3); and biomarkers of microbial translocation and inflammation (Aim 4) will be analyzed using the same approach described above. A secondary analysis will be conducted using a per protocol approach that includes only those participants who were adherent with their assigned intervention (i.e., taking zinc or placebo 80% of the time).

5.3 Missing Data

Enrollment and Attrition/Missing data: Participants who meet eligibility criteria and agree to participate will be compared with subjects who were determined to be eligible but declined enrollment on data captured during eligibility assessment. The 2 independent samples t-test and Fisher's exact test will be used to test for statistically significant differences between subjects who enroll and those who do not, and to test for significant differences between subjects lost to follow-up and those who complete it. Missing data patterns will be evaluated including the frequency and percentage of subjects missing for each variable and the distribution of the number of variables missing for subjects. In addition, data collected to the point of lost to follow-up will be compared to the data of those who complete the study to examine missing data mechanisms, e.g., missing completely at random (MCAR), missing at random (MAR), or not missing at random (NMAR). We will consider various approaches to account for missing data such as multiple imputation methods and likelihood-based approaches, if needed. In situations where missing data occurs, we will document the reasons for the missing data whenever possible. The proposed study has accounted for a 20% random noninformative loss to follow-up and will still have sufficient power with this potential loss in size. Multivariate multiple Imputation using iterative Markov Chain Monte Carlo (MCMC) method will be used to account for missing data . This model is the primary ITT analysis.

5.4 Additional Analyses

Effect of zinc over time: The main analyses will focus on the VACS index, our primary outcome, and other secondary outcomes at the 18 month time point as the primary interest is in evaluating the long-term effects of zinc and it is anticipated that effects will change over time. Additional analyses using generalized linear mixed effects models will be used to incorporate the repeated measures for each outcome in the same model and will test for possible zinc by time interactions (e.g. does the effect of zinc increase over time). The mixed effects models will include subject-specific

random intercepts and slopes to account for the correlation due to having repeated observations for each subject. The mixed models will be implemented in SAS PROC MIXED. In addition to the mixed effects model, we will also consider additional confirmatory models such as the general linear model for correlated data using an autoregressive covariance structure. We will use alpha=.10 to explore interaction. If the interaction is significant (i.e. p<0.10), subsequent stratified analyses will be conducted.

Moderators and Mediators:

Additional analyses will also be conducted to evaluate whether the effects of zinc depend on level of alcohol consumption. Models will be fit including a zinc by heavy alcohol interaction term. If the interaction is significant (i.e. p<0.10), subsequent stratified analyses will be conducted to evaluate the effect of zinc in each alcohol group.

Given the potentially complex role of ART use in the relationship between zinc and each outcome, various analyses will be considered to explore its possible role. First, it is not expected that initiation of ART use during follow-up to differ by randomized arm, however exploratory analyses will be conducted to compare initiation of ART use by randomized group. In addition, longitudinal analyses will be conducted including ART use as a time dependent variable in order to explore a potential zinc by ART use interaction (we will use alpha = 0.10 to test interaction). If ART use does not appear to be an effect modifier, we will also include it as time will also be included as a dependent covariate to evaluate whether it is a confounder. Lastly, to evaluate ART use as a potential mediator of the relationship between zinc and outcomes of interest, the approach described by Baron and Kenny will be used. To evaluate mediation, the change in magnitude of the zinc effect on our primary outcome, the VACS index (and our other secondary outcomes) in models with and without adjustment for ART use will be focused on. Results will be confirmed using structural equation models (SEMs) to simultaneously model the hypothesized mediated relationships. Fit statistics such as the root mean square error of approximation (RMSEA), Comparative Fit Index (CFI), and the chi-square statistic, will be presented to evaluate the goodness of fit of the SEMs.

To further assess initiation of ART, we will compare proportions initiating of ART by 18 months by randomized group using the chi-square test and also the logrank test to account for drop out/loss to follow-up.

We will also test zinc by heavy alcohol (past week) interaction.

We will test differences in VACS index components between groups.

5.5 Harms

The number of participants experiencing each AE/SAE will be presented for each treatment arm and categorized by severity and organ system. No formal statistical testing will be undertaken.

Causes of death will be presented by study arm and time to death will be analyzed using the log-rank test. Cox proportional hazards models adjusted for stratification factors will be used to estimate hazard ratios and 95% confidence intervals.

5.6 Statistical Software

Data will be analyzed with SAS version 9.4 (SAS Institute, Inc., Cary, NC).
