# Supplementary material for: Effect of Zinc Supplementation vs Placebo on Mortality Risk and HIV Disease Progression Among HIV-Positive Adults With Heavy Alcohol Use: A Randomized Clinical Trial
Source: JAMA Netw Open. 2020 May 8;3(5):e204330. doi: 10.1001/jamanetworkopen.2020.4330 (PMC7210486; doi:10.1001/jamanetworkopen.2020.4330)
Supplement: Supplement 2. — eTable. Causes of Death in the ZINC Trial by Randomized Group (N=33) [file jamanetwopen-3-e204330-s002.pdf]

Supplementary Online Content

Freiberg MS, Cheng DM, Gnatienko N, et al. Effect of zinc supplementation vs placebo on mortality risk and HIV disease progression among HIV-positive adults with heavy alcohol use: a randomized clinical trial. *JAMA Netw Open*. 2020;3(5):e204330. doi:10.1001/jamanetworkopen.2020.4330

eTable. Causes of Death in the ZINC Trial by Randomized Group (N=33)

This supplementary material has been provided by the authors to give readers additional information about their work.

© 2020 Freiberg MS et al. *JAMA Network Open.*

eTable. Causes of Death in the ZINC Trial by Randomized Group (N=33)

Cause of Death	***Zinc (N=21)***	***Placebo (N=12)***
HIV-related	*7*	*2*
Overdose	*4*	*1*
Pneumonia	*3*	*0*
Suicide	*2*	*0*
Unknown	*2*	*5*
Tuberculosis	*1*	*2*
Alcohol Poisoning	*1*	*0*
Thromboembolism	*1*	*0*
Traumatic Brain Injury	*0*	*1*
Influenza	*0*	*1*

© 2020 Freiberg MS et al. *JAMA Network Open.*
