# Supplementary material for: Effect of Zinc Supplementation vs Placebo on Mortality Risk and HIV Disease Progression Among HIV-Positive Adults With Heavy Alcohol Use: A Randomized Clinical Trial
Source: JAMA Netw Open. 2020 May 8;3(5):e204330. doi: 10.1001/jamanetworkopen.2020.4330 (PMC7210486; doi:10.1001/jamanetworkopen.2020.4330)
Supplement: Supplement 3. — Data Sharing Statement [file jamanetwopen-3-e204330-s003.pdf]

Freiberg MS, Cheng DM, Gnatienko N, et al. Effect of zinc supplementation vs placebo on mortality risk and HIV disease progression among HIV-positive adults with heavy alcohol use: a randomized clinical trial. *JAMA Netw Open*. 2020;3(5):e204330. doi:10.1001/jamanetworkopen.2020.4330

Data Sharing Statement

Data

Data available: Yes
Data types: Other (please specify)
Additional Information: Data collected for the study are available to interested investigators in the URBAN ARCH Repository: www.urbanarch.org.
How to access data: Data collected for the study are available to interested investigators in the URBAN ARCH Repository: www.urbanarch.org.
When available: With publication

Supporting Documents

Document types: None

Additional Information

Who can access the data: Data will be available to researches whose proposed use of the data has been approved.
Types of analyses: Data will be available to researches whose proposed use of the data and proposed analyses have been approved.
Mechanisms of data availability: Data will be released after approval of a proposal and after all appropriate IRB approvals have been received.
